# Eculizumab in atypical hemolytic uremic syndrome: strategies toward restrictive use

**DOI:** 10.1007/s00467-018-4091-3

**Published:** 2018-11-06

**Authors:** Kioa L. Wijnsma, Caroline Duineveld, Jack F. M. Wetzels, Nicole C. A. J. van de Kar

**Affiliations:** 1 Radboud Institute for Molecular Life Sciences, Amalia Children’s Hospital, Department of Pediatric Nephrology, Radboud University Medical Center, P.O. Box 9101, 6500 HB Nijmegen, The Netherlands; 2 Department of Nephrology, Radboud University Medical Center, Nijmegen, The Netherlands

**Keywords:** Atypical hemolytic uremic syndrome, Eculizumab, Restrictive treatment regimen, Personalized medicine, Thrombotic microangiopathy

## Abstract

With the introduction of the complement C5-inhibitor eculizumab, a new era was entered for patients with atypical hemolytic uremic syndrome (aHUS). Eculizumab therapy very effectively reversed thrombotic microangiopathy and reduced mortality and morbidity. Initial guidelines suggested lifelong treatment and recommended prophylactic use of eculizumab in aHUS patients receiving a kidney transplant. However, there is little evidence to support lifelong therapy or prophylactic treatment in kidney transplant recipients. Worldwide, there is an ongoing debate regarding the optimal dose and duration of treatment, particularly in view of the high costs and potential side effects of eculizumab. An increasing but still limited number of case reports and small cohort studies suggest that a restrictive treatment regimen is feasible. We review the current literature and focus on the safety and efficacy of restrictive use of eculizumab. Our current treatment protocol is based on restrictive use of eculizumab. Prospective monitoring will provide more definite proof of the feasibility of such restrictive treatment.

**Electronic supplementary material:**

The online version of this article (10.1007/s00467-018-4091-3) contains supplementary material, which is available to authorized users.

## Introduction

Over the last decade, the knowledge of the pathogenesis of atypical hemolytic uremic syndrome (aHUS) has increased substantially [1–3]. Atypical HUS is a rare and severe form of thrombotic microangiopathy (TMA) which predominantly affects the renal vasculature. The disease course is characterized by relapses, and both onset and recurrence are often triggered by a gastro-intestinal or pulmonary infection or pregnancy [1, 2]. Atypical HUS is caused by dysregulation of the alternative complement pathway, resulting in excessive production of the terminal complement complex C5b-C9, and subsequently endothelial cell injury. In up to 60% of the aHUS patients, pathogenic mutations in complement (regulatory) genes, such as complement factor H (CFH), membrane cofactor protein (MCP), and C3, can be detected. Additionally, about 10% of the patients, predominantly adolescents, have auto-antibodies directed against CFH [2]. Atypical HUS is associated with increased mortality, although the reported mortality varies in the different cohort studies. Mortality, largely dependent on the underlying pathogenic mutation, seems to be higher in children than in adults and could be estimated at 2–4% in adults and 8–14% in children at 3–5 years of follow-up [4, 5]. In the absence of effective treatment, approximately one third of the pediatric patients and half of adult patients with aHUS who did not die in the acute phase required, often permanent, dialysis treatment [4, 5]. The risk of recurrent disease after kidney transplantation was estimated to be 50 to 80%, with an overall 5-year graft survival of 36 ± 7% in patients with a recurrence compared with 70 ± 8% in patients without a recurrence [6–8].

Plasma therapy (PT), either plasma exchange (PE) or in some cases plasma infusions, became the cornerstone of treatment in aHUS in the early 1990s, despite the lack of randomized clinical trials and most information being based on retrospective studies with variable treatment protocols. In 2009, the European Pediatric Study Group for HUS was the first to publish a guideline to promote standardized high-volume and early initiated PT in aHUS patients [9]. The results of an audit analysis of this guideline were published in 2014 by Johnson et al. who evaluated 71 pediatric patients treated with PT [10]. Only 59 (83%) patients received some form of PT, and of these 59 patients, 13 received early high-volume PT as recommended by the guideline. Median (range) time until hematological remission in all patients was 11.5 days (0–119) and 83% of the patients reached hematological remission within 33 days. After 33 days, renal function had not fully recovered in most patients: 17% of the patients were still dialysis dependent, 46% suffered from persistent renal impairment, and 11% had either residual proteinuria and/or hypertension [10]. Of note, PT is not without risks: in most studies, various adverse events were noted, more in the pediatric population (up to 80%, especially young patients with low body weight had an increased risk) than in adults (26%), mostly catheter infections (50%) and thrombosis (19%) [1, 6, 10, 11].

By unraveling the role of complement in aHUS, novel therapeutic options emerged [12]. With the introduction of eculizumab in 2011, a new era was entered for treatment of aHUS patients. Eculizumab is a humanized, chimeric monoclonal antibody directed against complement component C5. By blocking the cleavage of C5 into C5a and C5b and subsequently the assembly of C5b-C9, endothelial injury is prevented [12]. Based on two prospective open-label phase-2 pivotal trials (including adults and adolescents) and a retrospective analysis of aHUS cohort (including pediatric, adolescent and adult patients) who received eculizumab outside the trials, eculizumab gained marketing approval and was adopted as first-line therapy in patients with aHUS [1, 12, 13]. In these trials, eculizumab was administered following a standard treatment scheme, which is currently recommended by the European Medicines Agency (EMA) and Food and Drug Administration (FDA) (Table 1). In the prospective studies, TMA event free status and complete TMA response was observed in respectively > 92 and 50–85% of the aHUS patients who were respectively resistant to PT or PT dependent [12]. PT could be discontinued in all patients. Overall, eculizumab treatment was well tolerated. Efficacy of eculizumab in the pediatric population was confirmed in a prospective open-label phase-2 trial conducted in 22 children below 18 years of age [14]. Complete TMA response was achieved after a median of 8.6 (1–22) weeks [14]. In the pivotal trials, eculizumab treatment was continued for at least 2 years [12, 13]. No advice was given on how to proceed when a patient was stable and in remission. Of note, TMA event free status and complete TMA response should not be interpreted as complete remission or full renal recovery (see also Table 6 in the manuscript of Loirat et al. for an extensive overview of definitions and attainment of endpoints) [1].

**Table 1:** Eculizumab dosage regimen, standard therapy according to EMA/FDA

Weight category	Induction phase	Maintenance phase
Above 40 kg	900 mg, every week, for 4 weeks	1200 mg, in fifth week, every 14 days thereafter
30 to < 40 kg	600 mg, every week, for 2 weeks	900 mg, in third week, every 14 days thereafter
20 to < 30 kg	600 mg every week, for 2 weeks	600 mg, in third week, every 14 days thereafter
10 to < 20 kg	300 mg once	300 mg, in second week, every 14 days thereafter
5 to < 10 kg	300 mg once	300 mg, in second week, every 31 days thereafter

Eculizumab has to be administrated intravenously

*EMA* European Medicines Agency, *FDA* Food and Drug Administration

Although these trials showed excellent results of treatment with eculizumab, the introduction of the drug initiated a worldwide debate regarding the optimal treatment strategy. Different questions were raised such as: what is the optimal duration of therapy? How can therapy be monitored? Is it safe to stop eculizumab therapy? Is there a need for prophylactic use of eculizumab in case of kidney transplantation? Guidelines, written by Kidney Disease Improving Global Outcome (KDIGO) or clinical recommendations generated by HUS international (a group of HUS experts), are inconclusive [1, 15]. This review will focus on the safety, effectiveness, and feasibility of restrictive eculizumab treatment.

## Eculizumab therapy: a none-ending story

Although no official document or international guideline directly addresses the duration of eculizumab therapy, it is assumed (and advocated in various scientific meetings and publications) that standard therapy is eculizumab in two weekly dosages lifelong [1, 15]. Indeed approval reports of both EMA and FDA emphasize the risks of withdrawal of eculizumab [15–17]. In most guidelines, both treatment duration and dosage of eculizumab are debated [1, 15]. There are reasonable arguments against the advised standard therapy.

First of all, there is little evidence to support lifelong therapy in every patient with aHUS. Before introduction of eculizumab, when PT was the mainstay therapy, renal outcome of aHUS patients was poor. However, some patients responded well to PT with hematological remission and recovery of kidney function and were not in need of chronic PT. Geerdink et al. evaluated a Dutch cohort of 45 pediatric aHUS patients [18]. Of these, 12 patients (25%) were not in need of chronic PT and did not relapse after the first aHUS episode. Fremeaux-Bacchi et al. reported 214 patients (89 children and 125 adults) with aHUS, of which 146 were treated with PT and followed for 4–5 years [4]. In 42% of the children and 34% of the adults, outcome was favorable; the remaining patients relapsed, reached end-stage renal disease (ESRD) after the first aHUS episode, or died. In 2006, Caprioli et al. reported the outcome of 60 aHUS patients with a mutation in CFH, MCP, or complement factor I (CFI). The majority of the patients was treated with PT for a period of 2 days to 6 weeks. After long-term follow-up renal function had normalized in 38% of the patients, including in 22.5% of the patients with a CFH mutation [19]. Jamme et al. evaluated the outcome of 156 adult aHUS patients treated with 5–20 sessions of PE. Overall outcome was poor as 14 patients died from aHUS or complications of treatment. After 1-year follow-up, renal function (according to Modification of Diet in Renal Disease equation (MDRD)) had recovered to an estimated glomerular filtration rate (eGFR) of ≥ 60 ml min^−1^ 1.73m^−2^ in 19% of the patients [20]. Some authors argue in favor of lifelong therapy while referring to the underlying genetic abnormality. However, many patients only present with disease in adulthood and have been free of disease in childhood despite contact with triggers such as vaccinations or infections [2].

Secondly, eculizumab treatment is not without risks. Although eculizumab is safe and well tolerated, potential (serious) adverse events need to be taken into account. The most prominent risk factor is the susceptibility to infections with encapsulated bacteria, especially meningococcal disease. By blocking the complement system, which is part of innate immunity, patients are more prone to infections with encapsulated bacteria, especially *Neisseria meningitidis*. The risk of invasive meningococcal disease is estimated at > 2000-fold increase compared with the normal population [21, 22]. To minimize the risk, patients are vaccinated against serotypes A, C, W, Y, and recently B and receive prophylactic antibiotics [21]. Furthermore, booster vaccinations every 5 years are highly recommended, since it is unknown what degree of protection is reached in patients with complement deficiencies [21]. Yet, a few cases have been described of fulminant meningococcal disease despite extensive vaccination and adequate antibody titers, questioning the effectiveness of the vaccines [23, 24]. The protective efficacy of vaccinations is less potent in patients who are treated with immunosuppressive drugs. Hence, a higher risk of infections can be expected in patients with aHUS after kidney transplantation. Furthermore, there is a risk of developing human anti-human antibodies (HAHA) which could neutralize eculizumab. Thus far, all case reports described non-neutralizing HAHA, which did not interfere with eculizumab efficacy [14] Other (long-term) chronic sequalae of eculizumab therapy are not yet fully comprehended. Only recently, emerging evidence appeared regarding the hepatotoxicity caused by eculizumab [25–27]. In retrospect, hepatotoxicity was already noted in ten patients during the pivotal trials as described in the approval package of the FDA [16]. Liver enzyme abnormalities were observed 10–29 days following initiation of eculizumab. In most cases, liver enzyme derangement was transient; however in some patients, eculizumab was withdrawn due to suspicion of drug-induced liver injury [25–27].

Thirdly, pharmacokinetic and pharmacodynamic data are only sparsely described in the pivotal trials and are insufficient to endorse the current treatment scheme [17, 28]. The recommended trough level of 50–100 μg ml^−1^ is based on a meta-analysis of patients with paroxysmal nocturnal hemoglobinuria (PNH) treated with eculizumab. Some PNH patients showed residual complement activity (based on a hemolytic assay to asses total complement activity (CH50)) at targeted eculizumab trough levels of 35 μg ml^−1^. Hence, trough levels of 50–100 μg ml^−1^ were advised for aHUS patients to minimize the risk of residual complement activation [17, 28, 29].

And last but not least, with costs up to €500,000 per year per patient, eculizumab is unaffordable in many countries or can only be afforded with specific restrictions. For example, The National Institute for Health and Care Excellence (NICE), located in the UK, stated in 2015 that eculizumab could only be reimbursed if the following arrangements were in place: coordination of eculizumab use through an expert center, a registry to monitor these patients, a national protocol with start and stop criteria and a research program to evaluate a restrictive treatment regimen [30, 31]. These criteria are under development and have not been published.

In this review, we discuss the available evidence and address the unanswered questions regarding eculizumab therapy, its prophylactic use, treatment dose, and duration of treatment. Prospective studies are needed to answer the open questions. Prospective evaluation of our current treatment protocol will provide some answers and expand the evidence.

## Strategies toward a restrictive use of eculizumab in patients with native kidneys

### When to start eculizumab therapy?

Early initiation of treatment (< 24–48 h) is highly recommended to stop TMA activity and to prevent chronic sequelae [1, 10, 32]. In pediatric patients, treatment with eculizumab is the preferred option, also in view of the high complication rate of PE in children [10, 11]. In adults, initial therapy with PE for 5 days is recommended. This will allow a detailed diagnostic work-up to exclude secondary causes of TMA, such as hypertension, drugs, or auto-immune diseases. This is in agreement with the pivotal study protocol, which included patients with progressive TMA after four or more sessions of PE and showed favorable outcomes. Of note, actual median (range) time to start eculizumab therapy in PE resistant patients was 0.8 months (0.2–3.7) in this study [12, 33]. A similar approach may also be feasible in adolescents, who have high prevalence of CFH auto-antibodies.

If the suspected diagnosis of complement-mediated aHUS is not refuted and patient did not respond to PE, eculizumab therapy should be initiated. Of note, TMA activity may disappear either spontaneously or with PE only in some patients with aHUS. In the latter cases, eculizumab therapy is not needed and PE can be gradually withdrawn as described by the European Pediatric Study Group for HUS [9, 10].

### Can we reduce eculizumab dosage?

Following the summary of product characteristics, eculizumab should be administered as two or three weekly infusions during the maintenance phase (Table 1) [17]. The pivotal trials aimed at reaching a trough level between 50 and 100 μg ml^−1^ considered necessary to fully block complement (CH50 < 10%). Therapeutic drug monitoring was not done, and all patients were treated with the advised fixed dose. It became clear that often serum eculizumab levels above target were reached, with reported trough levels up to 700 μg ml^−1^ in adults and up to 1100 μg ml^−1^ in children [16, 34]. This has stimulated clinical investigators to adapt the treatment schedule, by either increasing the interval or decreasing the dosage to maintain trough levels between 50 and 100 μg ml^−1^ [35]. Volokhina et al. evaluated 11 aHUS patients in whom treatment intervals were prolonged. Eculizumab concentrations ranged from 40 to 772, 61–367, 11–256, and 13–161 μg ml^−1^ after respectively 2, 3, 4, or 5 weeks interval. At intervals of 4–5 weeks, 80% of the patients had eculizumab trough levels > 50 μg ml^−1^. All patients with trough levels above 50 μg ml^−1^ had a fully blocked complement system as measured by CH50 (< 10%) [36]. Willrich et al. showed complete complement blockade with eculizumab trough levels of 100 μg ml^−1^, confirming the target levels as established in the pivotal trials [29]. Ardissino et al. reported that a median dose of 0.75 mg kg^−1^ day^−1^ (IQR 0.67–0.95) eculizumab was sufficient to block complement up to 4 weeks [37]. In comparison, the recommended dose of 1200 mg two weekly for a 70-kg adult provides 1.2 mg kg^−1^ day^−1^ and a 900-mg dose in a 40-kg child provides 1.6 mg kg^−1^ day^−1^. Gatault et al. developed a one-compartment model to predict pharmacokinetics and pharmacodynamics of eculizumab using the data of seven patients. Following their model, it would be possible to extend the interval to 4 weeks in patients less than 90 kg and even to 6 weeks in patients with body weight below 70 kg [35]. Of note, eculizumab trough levels are quite variable between patients with an inter-individual variation coefficient of 45% [35, 36].

The added value of therapeutic drug monitoring in patients treated with eculizumab is still unproven. Interpretation of eculizumab levels is difficult since the assays differ and all detect (to a variable degree) both bound and unbound eculizumab (Table 2). Instead of measuring eculizumab levels, the pharmacodynamic effect (total complement activity expressed as CH50) could also be used to monitor therapy. Most authors target a fully blocked complement system (CH50 < 10%). In contrast, a recent study evaluated efficacy of reduced dose of eculizumab, targeting CH50 < 30%. In a substantial amount of patients, this resulted even in less-effective complement blockade with CH50 between 10 and 70%. All 38 patients remained in remission [37]. Indeed, various studies reported relapse-free remissions in patients treated with eculizumab at extended intervals and incompletely blocked complement [36, 37, 47]. This suggest that complete complement blockade may not be necessary. Other markers of eculizumab activity and endothelial damage have been proposed. However, inconclusive and conflicting results have been published regarding the correlation between C3, C3d, C5, C5a, soluble C5b-C9, ex vivo endothelial cell assay, alternative pathway (AP50) activity, and efficacy of eculizumab therapy [36, 41, 42, 44]. Also, although treatment with eculizumab reduced the levels of markers of endothelial damage, their value in clinical practice is not proven [48].

**Table 2:** Monitoring of eculizumab therapy and complement activity in atypical hemolytic uremic syndrome (aHUS)

Parameter	Interpretation	Remarks
Serum eculizumab level	Target is set at trough levels of 50–100 μg ml^−1^ to fully block complement	Reports differ regarding the measurement of only the free proportion of eculizumab [36] versus measurement of eculizumab both free and bound to C5 [12, 14]. Furthermore, eculizumab can bind a maximum of 2 C5 molecules per eculizumab molecule and is able to bind both C5 as C5b incorporated in C5b-C9 complex. Hence, measurement of eculizumab can comprise free (excess) eculizumab, eculizumab bound to 1 C5, eculizumab bound to 2 C5, or in combination with C5b-C9 complexes. [38]. The assay used in the trials to determine the trough levels measured both bound and free proportion. [16, 39]
Eculizumab-C5 complex	In contrast with serum eculizumab levels, one could also determine eculizumab bound to C5, hence only the bound proportion of eculizumab is determined.	It is known that eculizumab can also bind C5b-C9. Furthermore, one eculizumab molecule could bind respectively 1 or 2 C5 molecules, hence the remaining capacity is unknown [40]. Clinical use is unknown.
Total complement activity (CH50)	CH50 levels correlated nicely with eculizumab serum trough levels, and suppressed CH50 (< 10%) is reached with trough levels > 30–50 μg ml^−1^ [28, 36, 39, 41]	There are different assays to measure CH50. With this test, total complement activity (also known as CH50) is tested to determine the capacity of patient serum to lyse sheep or chicken erythrocytes coated with antibodies. In the case of a functional complement system, the CP will be activated, consequently leading to C5b-C9 deposition on the erythrocytes and consequently cause hemolysis. With the Wieslab test, CH50 can be measured with C5b-C9 formation, detected using a C9 neoantigen, as read-out [41]. Recently, Willrich et al. reported CH50 measurement during eculizumab treatment with liposome immunoassay with stable and reliable results [29].
Alternative pathway activity (AP50)	AP50 levels can be suppressed by eculizumab, however ongoing activation has been noted despite adequate eculizumab levels [41, 42].	There are different assays to measure AP50. Specific assessment of alternative pathway activation is possible with a hemolytic assay based on untreated rabbit erythrocytes (AP50). Puissant-Lubrano et al. compared both the hemolytic assays as used in all trials with the Wieslab ELISA in 16 patients treated with eculizumab and found conflicting results [41]. Residual activity of the AP was observed with the hemolytic assay, in the presence of (sufficient) eculizumab levels. In contrast with the Wieslab ELISA which showed complete blockade of the AP. Moreover, they assessed sensitivity of all assays and concluded that the Wieslab ELISA is less sensitive, hence the residual activity measured with the hemolytic assay is most accurate [41].
C3d	C3d is a breakdown product of C3, hence elevated C3d complement levels reflect activation at level of C3 is present.	C3d levels are elevated in acute phase of aHUS and decreased in the majority of patient with eculizumab therapy [42, 43].
C3	Can be both normal as decreased in aHUS patients during acute phase and remission	[44]
C5	Eculizumab binds to C5. Eculizumab trough levels correlate with C5 levels.	C5 levels fluctuate between and within patients due to among others disease activity [29].
C5a	C5a is released after cleavage of C5. In the case of eculizumab therapy, C5 cannot be cleaved, hence less C5a is present.	Interestingly, values of C5a do not decrease to zero, although no C5a should be present in light of sufficient eculizumab [42]. Furthermore, C5a has a very short half-life of approximately 1 min.
Ex vivo endothelial cell assay	By determining the C5b-C9 deposition after adding patient serum on activated endothelial cells, complement blockade could be assessed with good reproducibility	This assay has one major drawback since it is a highly specialized technique which cannot be easily performed in any laboratory [44]. Although Noris et al. advocates that persistent complement activation tested ex vivo (whereas CH50 remained low) is a reason to increase eculizumab dosage or decrease interval, Merril et al. showed different results using a ham test [44, 45].
Ham test	The Ham test is modified from the assay used to detect PNH. By acidifying the patient serum, AP is activated and results in erythrocyte lysis in PNH. In the modified Ham test, PNH-like cells are incubated with serum of aHUS patients and depending on AP dysregulation present in the serum, will be lysed [46].	Merril et al. showed no correlation was seen between positive or negative Ham test, hence the presence of complement activation and aHUS recurrence. Moreover, various patients remained positive with the Ham test without disease recurrence and withdrawal of eculizumab therapy [45].
Soluble C5b-C9 (TCC)	Soluble C5b-C9 should decrease during eculizumab therapy	Various studies report different results. Due to the ability of eculizumab to bind C5b-C9, it could be possible that these complexes have a lower clearance, hence are elevated during remission [29, 36, 41, 42, 44].

*AP* alternative complement pathway, *CP* classical complement pathway, *ELISA* enzyme-linked immunoabsorbent assay, *PNH* paroxysmal nocturnal hemoglobinuria, *TCC* terminal complement complex

### Is withdrawal of eculizumab therapy possible?

Evidence to support lifelong therapy, as suggested shortly after the introduction of eculizumab is limited [12, 15]. In the past years, an increasing number of case reports and small cohort studies have provided information on eculizumab withdrawal. Nine reports have summarized the data of both children and adult patients in which therapy was either tapered and/or withdrawn (Table 3). In these studies, eculizumab was withdrawn in 171 patients after a median (range) of 6 months (0.5–50). Median (range) follow-up was 12 months (0–47). In the individual studies, relapse rate ranged from 20 to 67% [31, 45, 50–54]. Overall, 44 (27%) patients developed disease relapse. The median (range) time to relapse was 3 months (1–29.5). This is in agreement with earlier reports, dating from the pre-eculizumab era, indicating that 57–82% of relapses occurred in the first year of follow-up [4]. Due to close monitoring (among others screening for proteinuria and hematuria and rigorous control of blood pressure) and rapid re-initiation of eculizumab at the time of relapse, chronic sequelae could be prevented [31, 45, 50–54]. Obviously, the abovementioned studies have a relative short follow-up time of 12 months. Furthermore, they could be biased, since eculizumab was withdrawn in selected patients and not per protocol. Also, the duration of eculizumab therapy before withdrawal was quite variable. This selection process could have led to a more favorable outcome. However, a comparable relapse rate was noted in a study where eculizumab therapy was stopped per protocol at 3–6 months after start of therapy in adolescent and adult patients in remission [54]. In pediatric patients, eculizumab could be stopped in four patients and tapered in the remaining two children. A recurrence developed in one out of the six pediatric patients. Still, long-term follow-up is needed to provide more information regarding chronic sequelae of treatment withdrawal. It is also important to study possible predictors of relapse. In this respect, information regarding the presence and type of genetic mutation could be relevant.

**Table 3:** Studies describing a restrictive eculizumab regimen in aHUS patients

Author and year of publication	Number of participants	Age (years)	Duration standard eculizumab therapy (months)	Number of participants with tapered therapy	Number of participants in whom therapy was discontinued	Follow-up period after therapy adjustment (months)	Recurrence, number (%)	Time until recurrence (months)	Outcome
Cugno et al. (2014) [49]	18	Mean 21 (range 2–40)	Up till 40 months	18; interval was extended up to 4 weeks based on CH50	0	Up till 43 months	0	NA	No chronic sequelae
Ardissino et al. (2014–2015) [50, 51]	22	18 (1–53)	4.3 months (0.5–14.4)	0	16	Up till 40 months	5/16 (31%)	1.2 months (0.7–16.3)	No chronic sequelae after restart of eculizumab. Serum creatinine and proteinuria returned to baseline values
Sheerin et al. (2016) [31]	43	Unknown	6 months (0.5–8.5)	0	12	12 months	3/12 (25%)	2.5 months (1.5–9)	Full renal recovery was seen in 1 patient. The remaining 2 patient were still dialysis dependent on time of withdrawal and presented with hemolysis and hyperkalaemia which resolved quickly with reintroduction of eculizumab.
Fakhouri et al. (2017) [52]	108	30 (2–79)	17.5 months (2–50)	0	38	22 months (5–23)	12/38 (31%)	7.5 months (3–29)	No chronic sequelae after restart of eculizumab
Merril et al. (2017) [45]	17	46 (19–69)	3 months (0.5–18.2)	NA	15	10.2 months (1.2–46.3)	3/15 (20%)	2 months (1.8–3.3)	2 patients received eculizumab after which kidney function was restored. 1 patient died during PE for recurrence after non-adherence with antihypertensive drugs.
Macia et al. (2017) [53], summary of authors’ case reports	6	37 (16–39)	6 months (1–14)	1; patient received 900 mg every 4 weeks	5	Unknown	4/6 (67%)	3 months (2–12)	Unknown
Wijnsma et al. (2017) [54]	20	28 (1–62)	3.8 months (1.3–14.7)	5	15	27.4 months (6–47)	5/20 (25%)	7.5 months (1–12)	No chronic sequelae after restart of eculizumab. 1 relapse occurred during tapering eculizumab.
Ardissino et al. (2017) [37]	47	25 (0.5–60)	2.6 months (0.4–24.6)	38	9	26.9 months (0.8–80.9)	0	NA	No chronic sequelae after restart of eculizumab.
Macia et al. (2017) [53]; summary of clinical series	130	26 (0–80)	6.3 months (0.2–53.7)	0	61	5.6 months (0–35.1)	12/61 (20%)	3 months (1–29.5)	Limited data available. 1 patient progressed to ESRD despite re-initiation of therapy.

Numbers are expressed as median (range) unless otherwise specified. The different cohort studies and case reports include patients with atypical hemolytic uremic syndrome (aHUS) after kidney transplantation and patients with aHUS due to auto-antibodies directed against complement component factor H

*ESRD* end-stage renal disease, *NA* not applicable, *PE* plasma exchange

Due to still limited data on eculizumab withdrawal in children and adults with various pathogenic mutations, evaluating data deriving from the pre-eculizumab era is the best alternative up till now to estimate risk of relapse. Several large cohort studies have been published which looked at disease relapse in patients with different pathogenic mutations [4, 5]. Patients were treated with various protocols of PE or received only conservative therapy. In children and adults, the risk of relapse was estimated at 43 and 35%, respectively, mostly within the first year after presentation [4, 5]. The risk of recurrence seemed to be higher in patients with pathogenic mutation in CFH (31–55%), MCP (18–52%), and C3 (50%) [4, 5]. Long-term outcome in general was more favorable, despite the frequently reported relapses, in patients with a mutation in MCP [31, 45, 50–54]. Before the introduction of eculizumab, relapse rates were approximately 30% in patients without a known pathogenic mutation [4].

The risk of relapse in the nine mentioned studies is 27% after withdrawal of eculizumab. This compares favorably with the abovementioned relapse rates. Notably, since the introduction of eculizumab, to our knowledge, only one case of disease relapse after eculizumab withdrawal is reported in a patient without a proven pathogenic mutation [31]. In contrast, the risk of relapse is reported to be higher (up to 75%) in patients with a genetic variant in CFH [52]. In patients with a MCP mutation, a relative high risk up till 50% is reported after eculizumab withdrawal [52]. Our own data showed a relapse rate of 50% in patients with a CFH mutation, which is lower than previously reported in literature [54]. Limited data are published regarding the relapse rate after discontinuation of eculizumab in children, which ranges between 16 and 50% [9, 10]. In conclusion, the risk of relapse after eculizumab withdrawal is estimated at 30% and most relapses occurred within the first year after withdrawal. Pathogenic mutations in CFH and MCP seem to be associated with higher chance of recurrence. In contrast, withdrawal of eculizumab in patients without a proven pathogenic mutation was associated with a low risk of disease recurrence. In all reported cases of relapse, rapid re-initiation of eculizumab treatment allowed remission of aHUS and full renal recovery [52, 54].

Future studies should provide more insight in the relation between specific pathogenic mutations and risk of relapse and chronic sequelae.

### Is withdrawal of eculizumab therapy possible after relapse of aHUS?

There are very limited data on eculizumab withdrawal in patients with relapse of disease. In total, 17 patients were described who had a recurrence after eculizumab withdrawal. In three patients, with respectively pathogenic mutations in CFH, MCP, and C3, eculizumab was again discontinued after 3 to 20 months of treatment. No relapses have been reported [52, 54]. This suggests that even in a proportion of patients with disease recurrence, lifelong treatment is not necessary. However, future studies should provide more insight in the risks of multiple relapses and ability of eculizumab therapy to prevent chronic damage when given “on demand” (i.e., during active TMA episodes).

## Strategies toward a restrictive use of eculizumab in kidney transplant recipients

Kidney transplantation is associated with an estimated risk of aHUS recurrence of 50 to 80% [6, 55, 56]. Most recurrences occur early in the post-transplant period. Recurrent aHUS usually leads to graft loss and curative PE does not improve graft survival [8, 55]. The risk of recurrence depends on the genetic variant, and especially patients with a pathogenic mutation in genes that encode circulating complement components, such as CFH, CFI, complement factor B (CFB), or C3, have a high risk of recurrence (50–80%). In contrast, the recurrence rate is low (8%) in patients with a single genetic variant in MCP, a membrane-bound factor. This is not unexpected since the endothelium of the graft expresses a functional variant [6, 55, 56]. In patients without a pathogenic mutation, the risk of recurrence is reported to be moderate (18–28%) [5, 19]. A lower percentage of aHUS recurrence (20%) and graft failure was reported in children [57, 58], however, presumably in most of these studies patients with STEC-HUS were included. [59–62]. Sellier-Leclerc et al. more accurately diagnosed aHUS and reported a higher recurrence rate of 53% among pediatric patients, and even of 80% in patients with mutation in CFH. Graft survival at 1 year was 62% [63]. The recurrence risk is influenced by transplantation-related factors such as pre-donation kidney injury, ischemia reperfusion injury, acute rejections, infections, the use of mammalian target of rapamycin (mTOR) inhibitors, and calcineurin inhibitors (CNI) [55, 56, 61, 64, 65].

### Prophylactic eculizumab therapy in kidney transplantation

#### Effectiveness of prophylactic eculizumab therapy

The reported high recurrence rate and absence of effective treatment urged many transplantation centers to be very restrictive with offering a kidney graft to aHUS patients with ESRD. After the introduction of eculizumab, many centers started to perform kidney transplantation using eculizumab prophylaxis in various different schemes [66]. The first case series of aHUS patients treated prophylactically with eculizumab was published by Zuber et al. in 2012 [66]. Since then, in total, 53 patients, including 14 pediatric patients have been reported in literature (Online resource Table 1). As expected, the overall outcome of transplantation with prophylactic eculizumab therapy was favorable. In most patients, allograft function remained well preserved. Median (range) serum creatinine values, available in 38 patients, was 88 μmol l^−1^ (44–187) at 15 months (1.5–44 months) after transplantation. Four patients had signs of TMA after transplantation, which resolved after increase of eculizumab dose [31, 67–69]. One patient lost its allograft due TMA and a renal artery thrombosis, while on eculizumab therapy. No biopsy was performed [70].

#### Discontinuing prophylactic eculizumab therapy

In the patients who were treated prophylactically, eculizumab was discontinued in eight, all adults, at 1 to 28 months after transplantation [50, 53, 71–74]. Two patients, both recipients of a living donor kidney, developed aHUS recurrence after discontinuation of eculizumab (Online resource Table 1) [53, 74]. The remaining patients did well, without aHUS recurrence and serum creatinine ranged from 67 to 118 μmol L^−1^ after a follow-up of 4 to 26 months (Online resource Tables 1 and 4). These data indicate that continued eculizumab treatment is not needed in all adult patients after kidney transplantation.

### Kidney transplantation in aHUS patients without eculizumab prophylaxis

The reported high incidence of recurrent aHUS after kidney transplantation was based on studies that mainly included patients who received a deceased donor kidney, were treated with high-dose CNI, and often experienced rejection episodes. Small studies suggested better outcomes after living donor kidney transplantation [71, 75, 76]. Based on these data, we hypothesized that kidney transplantation without eculizumab prophylaxis should be feasible and developed a transplantation protocol to limit endothelial cell injury of the allograft and subsequent complement activation (Online resource Table 5). Initial data analysis showed a low aHUS recurrence rate [76]. Meanwhile, we have transplanted 19 adult patients with a living kidney donor. Eighteen patients had a high risk of recurrence according to the KDIGO classification; in seven patients, a mutation in the gene-encoding CFH was present. After a median follow-up of 42 months (4–78), two patients (one patient with a CFH mutation and one patients with a C3 mutation) developed aHUS recurrence, respectively after 2 and 4 months after transplantation, for which eculizumab was restarted. In these 19 patients, median (range) serum creatinine concentration at last follow-up is 138 μmol l^−1^ (79–185).

#### Effectiveness of rescue therapy with eculizumab

The pivotal trials included 25 patients who received eculizumab for aHUS recurrence after kidney transplantation. The median (range) interval between onset of aHUS recurrence and start of eculizumab was relatively long: 1.25 months (0.03–36.7). In 88% of the patients, a TMA event-free status was reached. In 20 patients who completed the 18-month follow-up, mean eGFR was 44 ml min^−1^ 1.73m^−2^ (SD 27) [77]. After an extended follow-up, two patients lost their grafts: one never responded to eculizumab and the second reportedly experienced TMA after a reduction in eculizumab dosage leading to ESRD [53]. In addition, we identified 56 patients, including five children (5,10, 13, 15, and 17 years old), from literature who had been treated with rescue therapy (Online resource Table 2). Patients with a known genetic mutation more often had experienced a recurrence in a previous allograft (online resource Table 3). In the majority of the patients (68%), the onset of recurrence was within 3 months after transplantation. The interval between the onset of aHUS and initiation of eculizumab varied between 0 and 279 days. Eculizumab was started within 7 days after onset of recurrence in only 22 (39%) patients. Of note, late initiation of eculizumab is associated with less recovery of kidney function [66]. Graft function was maintained in 47 (84%) patients. The remaining patients experienced graft loss despite rescue therapy (*n* = 9;16%) in three patients caused by aHUS of which one had received insufficient eculizumab treatment [71, 78, 79] and six patients lost their graft due to non-TMA-related causes (rejection, infectious complications, acute tubular necrosis) [71, 73, 78, 80–82]. Graft function after aHUS recurrence was reported in 38 patients: median (range) serum creatinine was 137 μmol l^−1^ (48–486) with a median (range) follow-up of 14 months (2–82) after onset of aHUS. Serum creatinine values exceeded 200 μmol l^−1^ in five patients [78, 83–86]. In all of them, eculizumab therapy had been suboptimal (late initiation of therapy or too early discontinuation) and/or renal function had been compromised before the onset of aHUS. We identified ten patients, with at least four patients [76, 87–89] at high risk of recurrence [15], in whom eculizumab was initiated within 28 days after onset of aHUS. Early treatment resulted in full recovery of renal function: median (range) serum creatinine was 113 μmol l^−1^ (53–159) before aHUS recurrence and 116 μmol l^−1^ (53–180) after treatment of recurrence [76, 87, 88, 90–95].

These data seemingly contrast with those reported by Legendre et al. These authors suggested that the recovery of kidney function in transplant patients treated with eculizumab was limited [77]. In a pooled post hoc analysis, which included 26 kidney transplant patients and 74 patients with a native kidney, recovery of kidney function was less pronounced in kidney transplant patients compared with patients with a native kidney: respectively a mean eGFR after 26 weeks of 37 ml min^−1^ 1.73 m^−2^ (SD 36), mean change from baseline 11 ml min^−1^ 1.73 m^−2^ (SD 20) versus 61 ml min^−1^ 1.73 m^−2^ (SD 41) and 38 ml min^−1^ 1.73 m^−2^ (SD 36) (*P* = 0.0092). These differences may partly be explained by the type of renal injury which differed between the groups: patients with aHUS in the native kidney had experienced a more rapid decrease of eGFR in the time interval compared with patients with post-transplant aHUS which showed a more gradual renal function deterioration. In addition, the time from aHUS diagnosis to introduction of eculizumab was in both patient groups much longer than currently accepted: in transplant patients median 1.25 months (range 0.03–36.7 months) versus 0.69 months (0.03–47.4 months) in patients with native kidneys. The above data suggest that rescue therapy with eculizumab in adult patients may result in acceptable recovery of renal function; however, early initiation of therapy is of paramount importance.

#### Discontinuing rescue therapy

In the abovementioned 56 patients eculizumab was discontinued in 17 adult patients (30%) (Online resource Table 2). A new recurrence occurred in 11 of 17 patients (65%) [65, 71, 73, 79, 81, 82, 86, 90, 96]. The recurrence rate in this group is higher compared with the recurrence rate reported in patients who stopped eculizumab prophylaxis after transplantation (2/8 patients; 25%). Of note, in all patients (*n* = 4) with a mutation in the gene-encoding CFH discontinuation of eculizumab led to a recurrence (Online resource Table 3) [71, 73, 76, 79]. In 9 of 11 patients, eculizumab was restarted. In six patients, renal function improved, however not to baseline values [65, 71, 76, 86, 90, 96]. Graft failure occurred in the other three but was not attributed to aHUS by the authors [71, 73, 82]. In contrast, a remarkably lower recurrence rate was described by Macia et al. in 2017 [53]. In their study, 16 patients from the pivotal trials, who had been treated with eculizumab for aHUS recurrence after transplantation, discontinued therapy. During a median follow-up of 24 weeks, one patient (without identified genetic variant) experienced aHUS recurrence for which eculizumab was restarted resulting in improvement of kidney function [77]. The high recurrence rate calculated from the case reports may be the consequence of publication bias. Otherwise, Macia et al. may have included several patients with de novo TMA after transplantation, not caused by aHUS, but by transplantation-related factors, explaining the low recurrence rate after discontinuation of eculizumab. Future studies are necessary to determine recurrence risk after discontinuation of rescue therapy and the consequences for renal outcome.

### Reduction of eculizumab dosage after kidney transplantation

No systematic data are available on the safety of tapering eculizumab therapy after transplantation to reach trough levels of 50 to 100 μg ml^−1^. However, patients have been described who were successfully treated with eculizumab at extended intervals [97–100]. In contrast, several patients developed aHUS activity after interval prolongation [66, 101]. Furthermore, patients have been reported with TMA while on standard maintenance therapy [31, 67–69]. In two of the latter cases, eculizumab trough levels were above target range and/or complement was completely blocked [67, 69]. Therefore, it has been suggested that higher eculizumab levels are necessary in patients after kidney transplantation when exposed to strong triggers of complement activation [69]. Alternatively, other causes of TMA in kidney transplant patients, such as infections, antiphospholipid antibodies, antibody-mediated rejection, or immunosuppressive drugs, must be considered in these situations.

## Monitoring

After therapy withdrawal, strict monitoring is essential, therefore we would recommend to see the patient regularly in the outpatient clinic under supervision of a physician with expertise in aHUS (Table 4). Since signs indicative of a subclinical or early phase of TMA can be subtle, and aHUS is a rare disease, it can be difficult to recognize and adjust treatment appropriately. Especially in kidney transplant recipient, aHUS can present as a smoldering disease. An allograft biopsy may disclose only subtle changes, mostly limited to swelling of vascular endothelial cells in capillaries and small arterioles [64].

**Table 4:** Monitoring disease activity in atypical hemolytic uremic syndrome (aHUS) patients

Characteristics aHUS	Regular workup during eculizumab therapy^a^	Regular workup after therapy withdrawal	Recurrence aHUS^b, c^
1. (Acute) Kidney injury	Serum creatinine	Serum creatinine	Serum creatinine greater than upper limit of normal per age or increase of > 15% compared with baseline
	Proteinuria (protein–creatinine ratio)	Proteinuria (protein–creatinine ratio)	Increase of > 25% in proteinuria
		Dipstick analyses twice per week at home	
	Bloodpressure (aim for P50)	Blood pressure measurement twice per week at home	NA
2. Thrombocytopenia	Platelets	Platelets	Platelet count < 150,000 × 10^3^ μl
3. Mechanical hemolytic anemia			Mechanical hemolysis is defined by the presence of at least 2 or more of the following criteria
	Hemoglobin	Hemoglobin	Below lowest limit of normal per age
	LDH	LDH	Greater than upper limit of normal
	Haptoglobin	Haptoglobin	Undetectable
	Schizocytes	Schizocytes	Appearance of schizocytes

After therapy withdrawal strict monitoring is essential. Regular workup after at least 1,2, 3, 6, 9, and 12 months is required. We would advise to monitor blood pressure at home. We aim for blood pressures around P50 for height and age (children) or < 130/80 mmHg (adults). Urine dipstick analysis at home could be considered, especially in children. Moreover, comprehensible instructions to the patient (and caregivers) when and how to contact their treating physician are essential. In case of signs indicating atypical hemolytic uremic syndrome (aHUS) recurrence such as high blood pressure, petechiae, fatigue, oliguria, jaundice, or a possible triggering event-like infection, the patient has to seek contact immediately. Of note, recurrent aHUS after kidney transplantation can present as a smoldering disease, with a slow increase in serum creatinine without overt systemic hemolysis. A allograft biopsy may disclose only subtle changes, mostly limited to swelling of vascular endothelial cells in capillaries and small arterioles [64]

*LDH* lactate dehydrogenase, *NA* not applicable, *P50* median percentile for height and age, *TMA* thrombotic microangiopathy

^a^Consider to monitor liver enzymes in light of potential hepatotoxicity, especially in patients with pre-existing liver disease

^b^Recurrece of aHUS is defined by the occurrence of all three characteristics of aHUS; acute kidney injury, thrombocytopenia, and mechanical hemolytic anemia

^c^A kidney biopsy to detect (smoldering) TMA can be of additional value

## Time for a paradigm shift in the treatment of aHUS with eculizumab

**Fig. 1:**
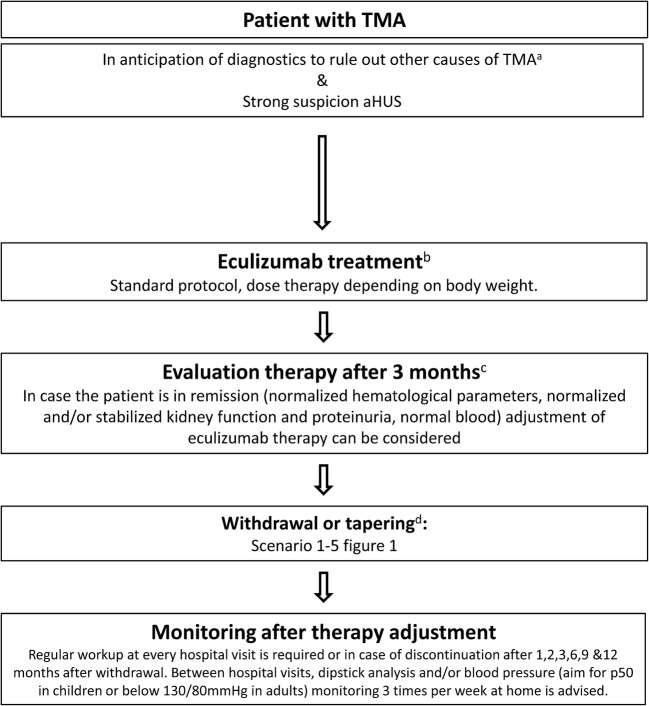
Treatment algorithm. After adequate exclusion of other causes of thrombotic microangiopathy (TMA) such as thrombocytopenic purpura (TTP), Shiga toxin-producing *Escherichia coli*-hemolytic uremic syndrome (STEC-HUS), or secondary TMA and in patients with strong suspicion of atypical hemolytic uremic syndrome (aHUS), eculizumab treatment should be started within 24 h after presentation. When the patient is stable and in remission, withdrawal or tapering can be considered, depending on patient characteristics (see Fig. 2). After therapy adjustment, strict monitoring is essential. NB in case of antibodies against complement factor H, a different treatment protocol has to be initiated as described by Loirat et al. [1]. a, For extensive overview of practical diagnostics approach for TMA, see Fakhouri et al. [3]. b, Treatment should preferably be started within 24 h after presentation. In adults with first episode of aHUS in native kidney, treatment with plasma exchange (PE) for 4 days (high volume PE with 1.5 plasma volume) is advised to allow diagnosis of secondary causes of aHUS. Adolescents may be considered adults [33]. After exclusion of secondary causes of aHUS and if the patient does not show a favorable response after 4 days of PE, treatment should be switched to eculizumab. Starting treatment with eculizumab within 7 days after presentation in PE-resistant patients was effective in the clinical trials [32]. In case the patient is PE sensitive, PE should be tapered and discontinued in the course of 1 month [9, 10]. c, Improvement of platelets and lactate dehydrogenase (LDH) is expected within 2–4 weeks. If no response, consider alternative diagnosis or inefficacy of eculizumab (C5 polymorphism p.Arg885His) [102]. d, See Fig. 2 for the different scenarios to withdraw or taper eculizumab, depending on patient characteristics

**Fig. 2:**
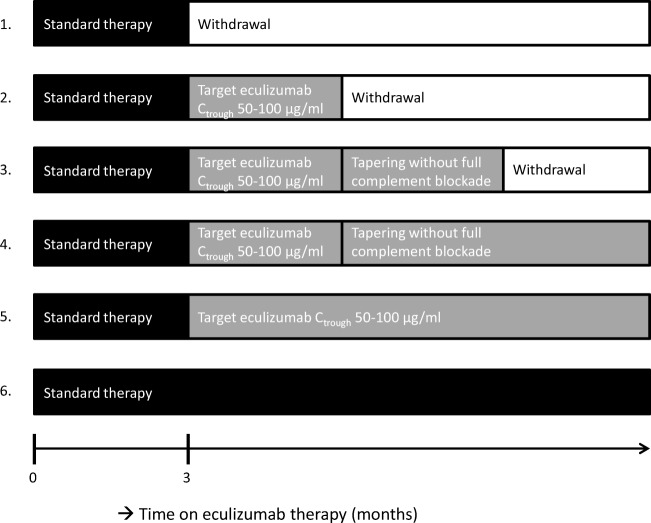
Scenarios for the treatment of atypical hemolytic uremic syndrome (aHUS) patients with eculizumab. The current standard regime is reflected by scenario 6, lifelong therapy with standard-dose eculizumab at biweekly intervals. Scenarios 1–5 illustrate options of restrictive eculizumab therapy. According to scenario 1, eculizumab is given at standard dose, at biweekly intervals, with withdrawal after 3 months. According to scenario 2, eculizumab dose will be adapted to target trough levels of 50–100 μg ml^−1^ with complete blockade of the complement system. After an observation period, eculizumab therapy will be withdrawn. In scenario 3, drug withdrawal is preceded by a period of eculizumab therapy at reduced dose and incomplete complement blockade (CH50 < 30%). For logistical reasons, dose reduction will be done by extending the dose interval. In scenarios 4 and 5, treatment with eculizumab will continue for an undefined period (“lifelong,” waiting for more data), either aiming at incomplete (scenario 4) or complete complement blockade (scenario 5). Of note, the observation periods are not strictly defined. The choice for a scenario as well as the length of the observation period will be dependent on patient characteristics, disease history, renal function, and patient or physician preferences. In patients with uncontrolled blood pressure, active (viral or bacterial) infection, reduced estimated glomerular filtration rate (eGFR) with evidence of continuous improvement (i.e., the nadir of serum creatinine has not been reached), or evidence of ongoing (extra-renal) thrombotic microangiopathy (TMA) activity, eculizumab treatment should be continued until stable remission is reached. Typical examples of the patient profiles which may best fit a proposed scenario are presented below. To aid the choice for a certain scenario a score can be calculated based on patient characteristics. The sum of the points will guide the selection of a scenario (online resource Table 6). Scenario 1: this is the proposed scenario used in adult patients with a first episode of aHUS in the native kidneys, who have adequately responded to treatment, with well-controlled blood pressure, stable renal function, and no signs of TMA. Scenario 2: this is the proposed scenario used in adults with a first relapse of aHUS in native kidneys, occurring more than 12 months after treatment withdrawal, who have adequately responded to treatment with recovery of eGFR, well-controlled blood pressure, and no signs of TMA. This scenario is also suitable for pediatric patients above 6 years of age and kidney transplant recipients with a relapse after transplantation with no pathogenic mutation. Scenario 3: this is the proposed scenario used in adults with first relapse of aHUS in native kidneys, occurring within 3–12 months after treatment withdrawal, who have adequately responded to treatment with recovery of eGFR, well-controlled blood pressure, and no signs of TMA. This scenario is also suitable for kidney transplant patients with a aHUS recurrence, successfully treated with eculizumab and pathogenic mutations in other genes than CFH. Scenario 4: this is the proposed scenario for patients with multiple relapses and kidney transplant recipients with a relapse (and CFH mutation) in which lifelong therapy is necessary. Scenario 5: this scenario is proposed for adult patients with relapse occurring during treatment with incomplete complement blockade, or with early (< 3 months) relapse after eculizumab withdrawal. This scenario is also used in pediatric patients below 6 years of age. They have an increased risk for frequent relapse since they are exposed to various infectious triggers during childhood. Therefore, withdrawal of eculizumab is not advised, but tapering of therapy to target serum trough levels could be beneficial to limit potential side effects and prevent overtreatment. Scenario 6: this scenario is proposed for patients with relapsing disease while receiving eculizumab therapy at doses targeted to levels of 50–100 μg ml^−1^. This scenario may also apply to patients with ESRD due to aHUS, with a history of graft failure due to disease recurrence, CFH mutations, or other high-risk factors. In these patients, any risk of recurrence should be avoided. These scenarios illustrate the treatment with eculizumab, as induction therapy for new-onset aHUS, either as first episode in incident patients or as relapse in prevalent patients. Prophylactic therapy with eculizumab in kidney transplant patients is not illustrated. We do not advise prophylactic therapy with eculizumab in each patient with aHUS. Still, we do not exclude the use of prophylactic therapy, in particular in children, in adult patient with a severe disease history, previous graft loss due to recurrence aHUS, genetic mutations in CFH, comorbidity (prior vascular events, known macrovascular disease), or highly sensitized patients. When prophylaxis is considered, we suggest to start with the induction dose 7–10 days before the kidney transplantation with a second dose 0–3 days before transplantation as eculizumab through levels of 50–100 μg ml^−1^ (and CH50 < 10%) may not yet be reached after the first dose. We would consider continued treatment with eculizumab after successful transplantation according to scenarios 4–6

Based on literature, we have developed a treatment protocol of restrictive eculizumab therapy (Fig. 1). Our protocol is founded on the principle that eculizumab can be withdrawn in patients with aHUS in remission after a first episode in native kidneys, routine eculizumab prophylaxis is not needed in adult patients before kidney transplantation, and that reducing the eculizumab dose is often possible in patients who need long-term therapy. Of note, decisions concerning individualization are based on age, disease history, co-morbidities, renal function, and patient and physician preferences. Many restrictive treatment strategies can be considered (Fig. 2). Obviously, the best strategy is currently unknown. Prospective studies are needed to allow comparisons and to compose evidence-based recommendations.

The Netherlands’ National Guideline was drafted based on our restrictive treatment protocol. The guideline was composed of the Dutch aHUS working group, comprising one nephrologist and one pediatric nephrologist from every academic hospital in the Netherlands. The guideline was approved by the professional societies and implemented in January 2016. In the Netherlands, several criteria must be met before orphan drugs can be (re)-imbursed by the National Healthcare Institute: the presence of an indication committee, the definition of clear start and stop criteria, monitoring of this process, and performing a cost-effectiveness analysis. In accordance with this policy start of eculizumab therapy in the Netherlands must be approved by the national aHUS working group (with four members being available on a daily basis) and treatment should follow the new guideline. Furthermore, a national prospective, observational study (abbreviated as CUREiHUS, NTR5988) was designed to monitor the guideline and eculizumab therapy. The study will be closed in August 2020. Analysis of our study data will provide evidence to develop optimized treatment protocols.

Because the optimal treatment strategy in aHUS is currently under investigation and our treatment scheme was designed as a study protocol, we recommend that all aHUS patients who are withdrawn of eculizumab will be included in a registry or national study to allow comparison and to aid future research. Furthermore, due to the rarity of aHUS and because treatment can be complicated, we believe monitoring of eculizumab therapy requires consultation with an aHUS referral center and access to a specialized laboratory capable of performing eculizumab and complement assays.

### Other studies of eculizumab discontinuation

To our knowledge, there are two other registered studies which evaluate safety and efficacy of eculizumab discontinuation. The first is a prospective study conducted in France (STOPECU, NCT02574403). The study protocol is well described, with criteria when and how to withdraw eculizumab. According to the protocol, eculizumab will be discontinued after 6 months of therapy. The primary end-point is the incidence of aHUS recurrence in a follow-up period of 2 years. The study will end inclusion in November 2019. The second study is conducted by Alexion Pharmaceuticals (EVIDENCE study, NCT02614898) with a planned end date of April 2020. This study is observational, without defined protocol, and will document TMA events and outcome of all included patients.

Since aHUS is a rare disease, and disease course is quite variable, we feel that a prospective study with inclusion of all treated patients and used of defined treatment algorithms is the best option to gain more insight in the pros and cons of different treatment options. We realize that withdrawal of eculizumab is not without risks. Still, lifelong therapy will expose many patients unnecessary to a drug which can cause side effects. Strict monitoring of the patients and rapid re-initiation of eculizumab therapy should limit risks. Obviously, the enormous costs of eculizumab cannot be disregarded. For a societal perspective, the health care budget is not infinite. Therefore, we must aim to develop cost-effective treatment strategies. The data provided by the CUREiHUS and STOPECU studies should help to develop cost-effective scenario’s which may vary per country depending on the local willingness to pay per quality-adjusted life year. We have calculated cost-effectiveness of prophylactic eculizumab therapy after kidney transplantation and showed that the costs exceeded the willingness to pay threshold in the Netherlands [103].

## Conclusions

Eculizumab therapy has changed the lives of patients with aHUS. The optimal treatment strategy is unknown. There is no evidence to support the need for lifelong therapy and untargeted treatment. Limiting the initial treatment period to 3 months in incident carefully evaluated patients with native kidney aHUS and withholding prophylactic therapy in patients with aHUS at transplantation is the first step toward restrictive use of eculizumab. Future studies, preferably at a multinational level, should evaluate the best strategies to prevent, predict, and treat relapsing disease. This includes targeted therapy aiming at complete and incomplete complement blockade and development of biomarkers that predict preclinical TMA activity.

## Electronic supplementary material

ESM 1 (DOCX 362 kb)
