# Correction to: Eculizumab in atypical hemolytic uremic syndrome: strategies toward restrictive use

**DOI:** 10.1007/s00467-018-4186-x

**Published:** 2019-01-15

**Authors:** Kioa L. Wijnsma, Caroline Duineveld, Jack F. M. Wetzels, Nicole C. A. J. van de Kar

**Affiliations:** 1 Radboud Institute for Molecular Life Sciences, Amalia Children’s Hospital, Department of Pediatric Nephrology, Radboud University Medical Center, P.O. Box 9101, 6500 HB Nijmegen, The Netherlands; 2 Department of Nephrology, Radboud University Medical Center, Nijmegen, The Netherlands

**Pediatric Nephrology**

10.1007/s00467-018-4091-3

The original version of this article unfortunately contained two mistakes. The presentation of Table 1 and Fig. 1 was incorrect. The corrected versions are given below.

**Table 1:** Eculizumab dosage regimen, standard therapy according to EMA/FDA

Weight category	Induction phase	Maintenance phase
Above 40 kg	900 mg, every week, for 4 weeks	1200 mg, in fifth week, every 14 days thereafter
30 to < 40 kg	600 mg, every week, for 2 weeks	900 mg, in third week, every 14 days thereafter
20 to < 30 kg	600 mg every week, for 2 weeks	600 mg, in third week, every 14 days thereafter
10 to < 20 kg	300 mg once	300 mg, in second week, every 14 days thereafter
5 to < 10 kg	300 mg once	300 mg, in second week, every 21 days thereafter

Eculizumab has to be administrated intravenously

*EMA* European Medicines Agency, *FDA* Food and Drug Administration

**Fig. 1:**
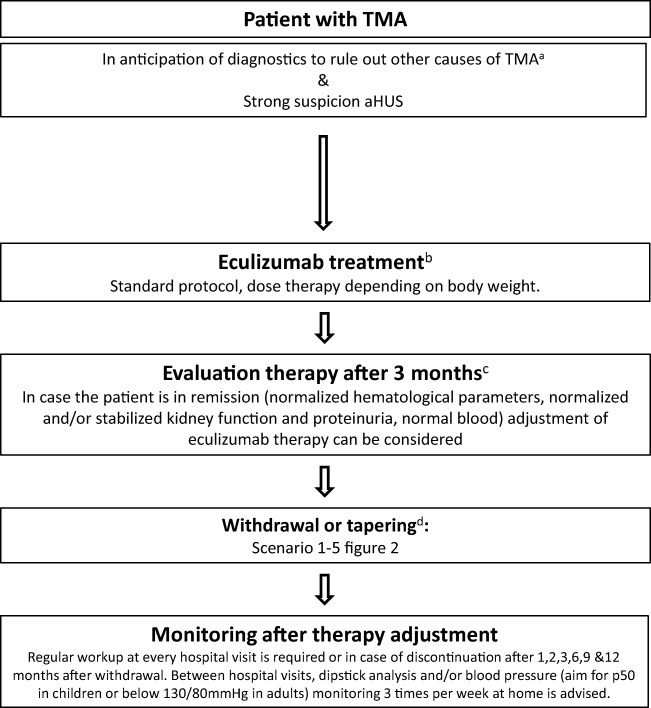
Treatment algorithm. After adequate exclusion of other causes of thrombotic microangiopathy (TMA) such as thrombocytopenic purpura (TTP), Shiga toxin-producing *Escherichia coli*-hemolytic uremic syndrome (STEC-HUS), or secondary TMA and in patients with strong suspicion of atypical hemolytic uremic syndrome (aHUS), eculizumab treatment should be started within 24 h after presentation. When the patient is stable and in remission, withdrawal or tapering can be considered, depending on patient characteristics (see Fig. 2). After therapy adjustment, strict monitoring is essential. NB in case of antibodies against complement factor H, a different treatment protocol has to be initiated as described by Loirat et al. [1]. a, For extensive overview of practical diagnostics approach for TMA, see Fakhouri et al. [3]. b, Treatment should preferably be started within 24 h after presentation. In adults with first episode of aHUS in native kidney, treatment with plasma exchange (PE) for 4 days (high volume PE with 1.5 plasma volume) is advised to allow diagnosis of secondary causes of aHUS. Adolescents may be considered adults [33]. After exclusion of secondary causes of aHUS and if the patient does not show a favorable response after 4 days of PE, treatment should be switched to eculizumab. Starting treatment with eculizumab within 7 days after presentation in PE-resistant patients was effective in the clinical trials [32]. In case the patient is PE sensitive, PE should be tapered and discontinued in the course of 1 month [9, 10]. c, Improvement of platelets and lactate dehydrogenase (LDH) is expected within 2–4 weeks. If no response, consider alternative diagnosis or inefficacy of eculizumab (C5 polymorphism p.Arg885His) [102]. d, See Fig. 2 for the different scenarios to withdraw or taper eculizumab, depending on patient characteristics

