# Supplementary material for: Eculizumab in atypical hemolytic uremic syndrome: strategies toward restrictive use
Source: Pediatr Nephrol. 2018 Nov 6;34(11):2261–77. doi: 10.1007/s00467-018-4091-3 (PMC6794245; doi:10.1007/s00467-018-4091-3)
Supplement: Supplementary file 1 — (DOCX 362 kb) [file 467_2018_4091_MOESM1_ESM.docx]

**Online resource table 1: prophylactic eculizumab therapy in patients with aHUS receiving a kidney transplantation**

| **Reference** | **aHUS in previous transplant** | **Recipients age (yrs) at Tx** | **Donor source** | **Reported genetic variant** | **Discontinuation of Ecu** | **Time (in months) on Ecu before discontinuation** | **Recurrence (time in months after Tx)** | **Restart of Ecu** | **Last FU (time in months after Tx)** | **Serum creatinine (µmol/L) (MDRD) at last FU** | **Remarks** |
| --- | --- | --- | --- | --- | --- | --- | --- | --- | --- | --- | --- |
| [1]Nieto-Ríos et al.2018 | No | 38 | DD | *CFH*: p. Arg257Cys | No | NA | No | NA | 18 | 71 |  |
| **[2]Macia et al.2017** | **No** | **38** | **LD** | **Negative** | **Yes** | **14** | **Yes (26)** | **Yes** | **Unknown** | **Unknown** | ¹ |
| [2]Macia et al.2017 | Unknown | 37 | LD | *MCP* | No | NA | No | NA | Unknown | Unknown |  |
| **[3]Krishnan et al.2017** | **No** | **54** | **LURD** | ***CFH*** | **Yes** | **12** | **Yes (17)** | **Yes** | **18** | **150** |  |
| [4]Manani et al.2017 | No | 45 | DD | *THBD*: p.Pro501Leu  *CFHR1-3* del with anti *CFH* ab | No | NA | No | NA | 7 | 81 |  |
| [5]Levi et al. 2017 | Unknown | 32 | DD | *CFI* | No | NA | No | NA | 4.4 | 124 |  |
| [5]Levi et al. 2017 | Unknown | 44 | DD | Anti *CFH* ab | No | NA | No | NA | 11.7 | 93 |  |
| [5]Levi et al. 2017 | Unknown | 37 | DD | *CFI*: p.Ile357Met | No | NA | No | NA | 14 | 157 |  |
| [5]Levi et al.2017 | Unknown | 56 | ABOi LD | Anti *CFH* ab | No | NA | No | NA | 14.7 | 133 |  |
| **Reference** | **aHUS in previous transplant** | **Recipients age (yrs) at Tx** | **Donor source** | **Reported genetic variant** | **Discontinuation of Ecu** | **Time (in months) on Ecu before discontinuation** | **Recurrence (time in months after Tx)** | **Restart of Ecu** | **Last FU (time in months after Tx)** | **Serum creatinine (µmol/L) (MDRD) at last FU** | **Remarks** |
| [5]Levi et al. 2017 | Unknown | 48 | DD | *CFI*: p.Gly243Val | No | NA | No | NA | 20.2 | 121 |  |
| [5]Levi et al. 2017 | Unknown | 39 | DD | *C3*: p.Arg161Trp | No | NA | No | NA | 21.9 | 97 |  |
| [5]Levi et al. 2017 | Unknown | 18 | DD | *CFH*: p.Asn767LysfsX7  *CFI*: p.His183Arg | No | NA | No | NA | 27.4 | 187 |  |
| [5]Levi et al. 2017 | Unknown | 40 | DD | Unknown | Yes | 28.7 | No | NA | 32.5 | 77 |  |
| [5]Levi et al. 2017 | Unknown | 38 | LD | *CFH*: p.Gln81Pro | No | NA | No | NA | 35 | 171 |  |
| [5]Levi et al. 2017 | Unknown | 50 | DD | *CFH*: p.Tyr1177Cys | No | NA | No | NA | 44.3 | 178 |  |
| [6]de Andrade et al. 2017 | Unknown | 29 | DD | *CFH* | No | NA | Yes, recurrence while on eculizumab (4) | NA | 4 | Graft loss | Graft loss (while experiencing endovascular problems) due to aHUS while on maintenance therapy (1200 mg, every 15 days) despite additional dosage of Ecu. |
| [6]de Andrade et al. 2017 | No | 17 | DD | *CFHR5*: p.Arg356His | No | NA | No | NA | 4 | 84 |  |
| **Reference** | **aHUS in previous transplant** | **Recipients age (yrs) at Tx** | **Donor source** | **Reported genetic variant** | **Discontinuation of Ecu** | **Time (in months) on Ecu before discontinuation** | **Recurrence (time in months after Tx)** | **Restart of Ecu** | **Last FU (time in months after Tx)** | **Serum creatinine (µmol/L) (MDRD) at last FU** | **Remarks** |
| [7]Sheerin et al. 2016  ***8 pts*** | Unknown | Unknown | Unknown | Unknown | No | NA | No | NA | Unknown | Unknown (“good transplant function”) | ² |
| [8]Sun et al.2016 | No | 42 | DBD | *CFH* | No | NA | No | NA | 6 | 66 |  |
| [9]Akchurin et al.2015 | No | 10 | LURD | *CFH*: p.Trp920Arg | No | NA | No | NA | Unknown | Unknown (“stable renal function”) |  |
| [10]Kasapoǧlu et al.2015 | No | 18 | LRD | *CFH*: p. Asp268Asn  *CFH*: p.Tyr271stop | Yes | 1 | No | NA | 12 | 67 |  |
| [11]Mallet et al.2015 | Yes | 18 | LRD | Negative | No | NA | No | NA | 29 | 116 |  |
| [12]Alasfar et al.2015 | Yes | 35 | DD | *CFH*: int20br-CFHR3  *CFH*: CFHR1int4-CFHR4 block 7/2 prime | No | NA | No | NA | Unknown | 44 |  |
| [12]Alasfar et al.2015 | No | 27 | LURD | *CFH* | No | NA | No | NA | Unknown | 97 |  |
| **Reference** | **aHUS in previous transplant** | **Recipients age (yrs) at Tx** | **Donor source** | **Reported genetic variant** | **Discontinuation of Ecu** | **Time (in months) on Ecu before discontinuation** | **Recurrence (time in months after Tx)** | **Restart of Ecu** | **Last FU (time in months after Tx)** | **Serum creatinine (µmol/L) (MDRD) at last FU** | **Remarks** |
| [13]Riddell et al.2015 | No | 33 | LURD | *CFH:* p.Arg1251Gly | No | NA | Yes, subclinical TMA in biopsy while on eculizumab (9) | NA | ± 24 | 150 | Incomplete complement blockade with 1200 mg Ecu every two weeks. Increase of dosage to 1500 mg every two weeks. |
| [14]Parikova et al.2015 | No | 43 | LUR | *CFH*: p.Ser451Stop | No | NA | Yes, TMA in peripheral blood (0.5) | NA | 18 | 150 | TMA in peripheral blood on day 14 despite therapeutic through levels of Ecu (293 µg/ml). Treatment scheme was intensified |
| [15]Ažukaitis et al.2014 | No | 6 | DD | *C3*: p.Pro1640Leu | No | NA | No | NA | 9 days after Tx | Patient deceased | Patient deceased due to iCVA possibly extrarenal manifestation of aHUS, no signs of peripheral blood TMA. |
| [16]Ardissino et al.2014 | No | 19 | Type unknown | Anti *CFH* ab | Yes | ± 5.5 | No | NA | 31.4 | 118 |  |
| [17]Ranch et al.2014 | No | 3 | DD | *CFH*: p.Ser1191Trp | No | NA | Yes, TMA in peripheral blood (2 days) | NA | Unknown | Unknown (“*stable kidney function”*) | Maintenance therapy with Ecu (dosage of 300 mg 11 days before and on day 0 of Tx). TMA in peripheral blood on day 2 after Tx for which treatment scheme was intensified. |
| **Reference** | **aHUS in previous transplant** | **Recipients age (yrs) at Tx** | **Donor source** | **Reported genetic variant** | **Discontinuation of Ecu** | **Time (in months) on Ecu before discontinuation** | **Recurrence (time in months after Tx)** | **Restart of Ecu** | **Last FU (time in months after Tx)** | **Serum creatinine (µmol/L) (MDRD) at last FU** | **Remarks** |
| [18]Román-Ortiz et al.2014 | No | 9 | DD | *CFH*: p.Ser890Ile  C*FH*: p.Val1007Leu  *CFH/CFHR1* hybrid gene | No | NA | No | NA | 36 | 80 |  |
| [19]Matar et al. 2014 | Yes | 51 | LRD | Negative | Yes | 6 | No | NA | 12 | 88 |  |
| [19]Matar et al. 2014 | No | 38 | LRD | Negative | Yes | 6 | No | NA | 12 | 97 |  |
| [19]Matar et al. 2014 | Yes | 40 | LURD | Negative | Yes | 6 | No | -NA | 12 | 115 |  |
| [19]Matar et al. 2014 | Yes | 27 | LURD | *CFH*: p.Ser1191L  *CFH*: p.Val1197Ala | No | NA | No | NA | 12 | 80 |  |
| [20] Békássy et al.2013 | Yes | 11 | DD | *CFI*: p.Gly261Asp  *CFB*: p.Leu433Ser | No | NA | No | NA | 12 | (79 ml/min/1.73m²) |  |
| [21]Pelicano et al.2013 | No | 27 | LRD | *CFH*: p.Pro116Leu | No | NA | No | NA | 15 | 76 |  |
| [22]Zuber et al.2012 | No | 17 | DD | *CFH-CFHR1* hybrid gene | No | NA | No | NA | 14 | 87 |  |
| **Reference** | **aHUS in previous transplant** | **Recipients age (yrs) at Tx** | **Donor source** | **Reported genetic variant** | **Discontinuation of Ecu** | **Time (in months) on Ecu before discontinuation** | **Recurrence (time in months after Tx)** | **Restart of Ecu** | **Last FU (time in months after Tx)** | **Serum creatinine (µmol/L) (MDRD) at last FU** | **Remarks** |
| [22]Zuber et al.2012 | No | 10 months | DD | *C3:* p.Arg161Trp | No | NA | No | NA | 4.5 | 44 |  |
| [22]Zuber et al.2012 | No | 1 | DD | *CFH:* Gln1137X | No | NA | No | NA | 4 | 58 |  |
| [22]Zuber et al.2012 | Yes | 3 | DD | *CFH:* p.Ser1191Leu | No | NA | No | NA | 1 day after Tx | Graft loss | Graft loss due to arterial thrombosis, transplant nephrectomy at day 3. |
| [22]Zuber et al.2012 | Yes | 33 | DD | *CFH:* p.Tyr1177Cys | No | NA | No | NA | 1.5 | 176 | 6 weeks after Tx mixed rejection. |
| [23]Xie et al.2012 | No | 31 | LURD | *CFH*: p.Gluc625Stop | No | NA | No | NA | 12 | 78 |  |
| [24,25]Krid et al.2012 | No | 7 | DD | *CFH-CFHR1* hybrid gene | No | NA | No | NA | 15 | 48 |  |
| [25,26]Nester et al.2011 | Yes | 12 | LURD | *CFH-CFHR1* hybrid gene | No | NA | No | NA | 16 | 70 |  |
| [25,27]Weitz et al.2011 | No | 7 | DD | *CFH:* p.Glu1189Stop | No | NA | No | NA | 23 | 44 |  |
| [25,28]Zimmerhackl et al. 2010 | No | 9 | DD | *CFH:* p.Trp1183Cys | No | NA | No | NA | 39 | 46 |  |

*Case reports and case series found in literature with prophylactic use of eculizumab in aHUS patients receiving a kidney transplantation. In several case reports serum creatinine (or follow up time) is not described in text but depicted in a figure, the approximate values were used in this table and designated as ± . Eight patients discontinued eculizumab after transplantation and are indicated in red. The patients printed in* ***bold*** *had a recurrence after discontinuation.*

*Abbreviations: LRD= living related donor; LD= living donor; DD= deceased donor; DBD= donation after brain death; DCD= donation after cardiac death; LURD= living unrelated donor; ABMR= antibody mediated rejection; CFH= complement factor H; CFI= complement factor I; CFB=complement factor B; C3= complement factor 3; MCP= membrane cofactor protein; THBD= thrombomodulin; CFHR= complement factor H related protein; del= deletion; Tx = kidney transplantation; MDRD = modification of diet in renal disease; ABOi= ABO incompatible; anti CFH ab= anti CFH antibodies; Ecu=eculizumab; NA= not applicable; Bx=kidney biopsy; TMA = thrombotic microangiopathy; PE = plasma exchange; GL =graft loss; ATN = acute tubular necrosis; GI = gastro-intestinal; IVIG = intravenous immunoglobulins; Scr= serum creatinine; HD = hemodialysis; iCVA = ischemic cerebrovasculair accident; FU = follow-up.*

*¹ after six months eculizumab was discontinued again, resulting in a recurrence 4 months later, possibly triggered by a urine tract infection. Eculizumab was restarted as lifelong therapy. The renal outcome is unknown.*

*² one patient with a CFH mutation showed deterioration of renal function, incomplete complement blockade and low-grade TMA on renal biopsy during treatment with eculizumab (1200 mg every two weeks). Eculizumab treatment was intensified to 1500 mg every two weeks. Trough levels of eculizumab are unknown. No details on the post-transplantation period of the other patients were reported.*

**Online resource table 2: efficacy of eculizumab rescue therapy in patients with aHUS after kidney transplantation**

| **Reference** | | **aHUS in previous transplant** | **Recipients age (yrs) at Tx** | **Donor source** | **Reported genetic variant** | **Time from Tx to recurrence** | **Time from recurrence to start of Ecu** | **SCr ( µmol/L) (MDRD) before aHUS** | **Scr (µmol/L) (MDRD) at start of ecu** | **Nadir Scr (µmol/L) after 1^st^ recurrence; follow-up (in months) after Tx** | **Discontinuation of Ecu** | **Second recurrence (time in months after Tx)** | **Restart ecu** | **Remarks** |
| --- | --- | --- | --- | --- | --- | --- | --- | --- | --- | --- | --- | --- | --- | --- |
| [29]Devresse et al. 2018 | No | 23 | LRD | *CFI:* p.Pro50Ala  *MCP*  *CFHR1-3* del | 40 days | ± 4 months | 107 | ±486 | 177; ± 17 | Yes | No | NA |  |  |
| [30]Zwang et al.2018 * | Unknown | 39 | DD | *CFI*: p. Ile398Leu  *CFHR1-3* del | 7 months | ± 15 days | 300 | 1043 | 486; 10 | No | No | NA | Poor renal function before aHUS recurrence due to persistent borderline rejection. |  |
| [31] Vondrak et al. 2018 | No | 13 | DD | *CFH/CFHR1 /CFHR3* hybrid gene | 2 months | 5 days | Unknown | 322 | 109; 84 | No | No | NA |  |  |
| [6]de Andrade et al.2017 | No | 30 | LD | Negative | 4 days | Max 2 days | Unknown | ¹ | 141; 42 | No | No | NA |  |  |
| [6]de Andrade et al. 2017 | Unknown | 20 | DD | *CFH*: p.Asn1050Tyr  *CFH*: del in exon 23  *CFRH1-3* del | 30 days | Max 2 days | Unknown | ¹ | 80; 42 | No | No | NA |  |  |
| **Reference** | **aHUS in previous transplant** | **Recipients age (yrs) at Tx** | **Donor source** | **Reported genetic variant** | **Time from Tx to recurrence** | **Time from recurrence to start of Ecu** | **SCr ( µmol/L) (MDRD) before aHUS** | **Scr (µmol/L) (MDRD) at start of ecu** | **Nadir Scr (µmol/L) after 1^st^ recurrence; follow-up (in months) after Tx** | **Discontinuation of Ecu** | **Second recurrence (time in months after Tx)** | **Restart ecu** | **Remarks** |  |
| [6]de Andrade et al.2017 | No | 36 | DD | Negative | 2 months | Max 2 days | Unknown | ¹ | 185; 7 | No | No | NA |  |  |
| [6]de Andrade et al.2017 | No | 32 | DD | *CFI*: p.Ile416Leu  *CFRH1-3* del | 1 month and 20 days | Max 2 days | Unknown | ¹ | 194; 9 | No | No | NA |  |  |
| [6]de Andrade et al.2017 | No | 44 | DD | Unknown | 1 day | Max 2 days | Unknown | ¹ | 141; 6 | No | No | NA | Patient died with functioning graft 6 months after transplantation due to aspergillus infection. |  |
| [5]Levi et al. 2017 | Yes | 26 | DD | *CFH*  *CFH/CFHR1* hybrid gene | Within 25 days | Unknown (Ecu started d25 after Tx) | Unknown | Unknown | Graft loss; 55 | Yes | Yes (56) | Unknown | Graft loss due to chronic graft dysfunction and sepsis, resulting in restart of HD. After subsequent withdrawal of Ecu aHUS recurrence. |  |
| **Reference** | **aHUS in previous transplant** | **Recipients age (yrs) at Tx** | **Donor source** | **Reported genetic variant** | **Time from Tx to recurrence** | **Time from recurrence to start of Ecu** | **SCr ( µmol/L) (MDRD) before aHUS** | **Scr (µmol/L) (MDRD) at start of ecu** | **Nadir Scr (µmol/L) after 1^st^ recurrence; follow-up (in months) after Tx** | **Discontinuation of Ecu** | **Second recurrence (time in months after Tx)** | **Restart ecu** | **Remarks** |  |
| [5]Levi et al. 2017 | Yes | 23 | DD | *CFH*: p.Ser1191Leu  *CFH*: p.Val1197Ala | Within 6 days | Unknown (Ecu started d6 after Tx) | Unknown | Unknown | NA | No | No | NA |  |  |
| [32]Munch et al.2017 | Yes | 58 | DD | *CFHR1-3* del  Anti *CFH* ab | 21 days | Unknown (after 6 sessions of PT) | Unknown | ± 270 (incomplete respons to PT) | ±150; 3.8 | Yes | Yes (4.7) | Yes | Scr after 2^nd^ recurrence ± 140µmol/L 18 months after Tx. |  |
| [33]Shochet et al.2017 * | No | 45 | DD (pancreas-kidney) | Unknown | 7 days | 17 days | 315 | HD | 267; ±2.3 | Yes | Yes (±2.8) | Yes | Renal function compromised due to infectious complications before and after transplantation. Scr after 2^nd^ recurrence ± 182µmol/L 6 months after Tx. |  |
| [34]Asif et al.2017 | No | 37 | LRD | Negative | 8 days | Unknown | 80 | HD | 150; 6 | No | NA | NA | - |  |
| **Reference** | **aHUS in previous transplant** | **Recipients age (yrs) at Tx** | **Donor source** | **Reported genetic variant** | **Time from Tx to recurrence** | **Time from recurrence to start of Ecu** | **SCr ( µmol/L) (MDRD) before aHUS** | **Scr (µmol/L) (MDRD) at start of ecu** | **Nadir Scr (µmol/L) after 1^st^ recurrence; follow-up (in months) after Tx** | **Discontinuation of Ecu** | **Second recurrence (time in months after Tx)** | **Restart ecu** | **Remarks** |  |
| [35] Duineveld et al. 2017 | Yes | 41 | LURD | *CFH:* p.Trp678Cys | 64 days | 1 day | 94 | 145 | 110; 5 | Yes | Yes (±5.2) | Yes | Possible recurrence after Tx with peripheral blood hemolysis without TMA in Bx or alternative cause TMA. Scr after 2^nd^ relapse 143µmol/L 14 months after Tx. ² |  |
| [36]Dedhia et al.2017 * | No | 28 | LURD | *CFHR1-3 del* | ± 40 days | 0 days | 221 | 486 | 132; 30 | No | No | NA | Poor renal function before aHUS recurrence due to rejection and bacteremia. |  |
| **Reference** | **aHUS in previous transplant** | **Recipients age (yrs) at Tx** | **Donor source** | **Reported genetic variant** | **Time from Tx to recurrence** | **Time from recurrence to start of Ecu** | **SCr ( µmol/L) (MDRD) before aHUS** | **Scr (µmol/L) (MDRD) at start of ecu** | **Nadir Scr (µmol/L) after 1^st^ recurrence; follow-up (in months) after Tx** | **Discontinuation of Ecu** | **Second recurrence (time in months after Tx)** | **Restart ecu** | **Remarks** |  |
| [2,37]Legendre et al. Macia et al. 2017  **25 pts** | Unknown | Median 41.5 (17-69) | Unknown | 13 (50%) with complement mutation or autoantibody | Unknown | 1.25 months (range 0.03-36.7 months) | Unknown | Median eGFR 22.2 ml/min/1.73m² (range 10-72.3) | Mean eGFR 44 ml/min/1.73m² (SD 27); 18 months after start of eculizumab | Yes, 16 pt | Yes, in 3 patients (including 1 patient during a dose reduction) | Yes, restart in 1 patient | In patient who restarted Ecu renal function improved. Graft loss occurred in the other two patients due to aHUS. A forth patient died, not due to aHUS. |  |
| [38]Yamamoto et al. 2017 | No | 35 | LD | Negative | 15 days | 22 days | ± 136 | Unknown | 116; 36 | Unknown | No | NA | Treated for ABMR before aHUS recurrence. |  |
| [7]Sheerin et al. 2016 | Unknown | Unknown | Unknown | Unknown | Early post-operative period | Unknown | Unknown | Unknown | Unknown ( “*good transplant function”)* | Unknown | No | NA | Further details are missing. |  |
| **Reference** | **aHUS in previous transplant** | **Recipients age (yrs) at Tx** | **Donor source** | **Reported genetic variant** | **Time from Tx to recurrence** | **Time from recurrence to start of Ecu** | **SCr ( µmol/L) (MDRD) before aHUS** | **Scr (µmol/L) (MDRD) at start of ecu** | **Nadir Scr (µmol/L) after 1^st^ recurrence; follow-up (in months) after Tx** | **Discontinuation of Ecu** | **Second recurrence (time in months after Tx)** | **Restart ecu** | **Remarks** |  |
| [7]Sheerin et al. 2016 | Unknown | Unknown | Unknown | C3 | 29 months | Unknown | Unknown | Unknown | Unknown ( “*improvement in transplant function”)* | Unknown | No | NA | Further details are missing. |  |
| [7]Sheerin et al. 2016 | Unknown | Unknown | Unknown | CFH | Early after Tx | Unknown | Unknown | Unknown | Unknown ( “*stable transplant function”)* | Unknown | No | NA | Further details are missing. |  |
| [7]Sheerin et al. 2016 | Unknown | Unknown | Unknown | Unknown | Early after Tx | Unknown | Unknown | Unknown | Unknown ( “*stable transplant function”)* | Unknown | No | NA | Further details are missing. |  |
| [39]Ikeda et al.2016 | No | 27 | LRD | Not investigated | 1 day | 2 days | Unknown | ± 681 | 117; 8 | Yes | No | NA | - |  |
| [39]Ikeda et al.2016 | No | 66 | LURD | Not investigated | 1 day | 5 days | Unknown | ± 575 | 120; 12 | Yes | No | NA | - |  |
| [40]Okumi et al.2016 | Nov | 30 | LRD | *CFH*: p.Arg1215Gln | 3 weeks | ± 8 months | 133 | Unknown | 158; 60 | No | No | NA | - |  |
| [41]Salameh et al.2016 | No | 48 | DD | *CFH*: p.Arg1215Gln | 3 months | Unknown (after 7 session of PT) | 133 | >353 | 133; 9 | No | NA | NA | - |  |
| [42]Juega-Marino et al.2016 | No | 46 | DD (pancreas-kidney) | Negative | 7 months | >10 days | 110 | 438 | 180; >10 | Yes | No | NA | - |  |
| **Reference** | **aHUS in previous transplant** | **Recipients age (yrs) at Tx** | **Donor source** | **Reported genetic variant** | **Time from Tx to recurrence** | **Time from recurrence to start of Ecu** | **SCr ( µmol/L) (MDRD) before aHUS** | **Scr (µmol/L) (MDRD) at start of ecu** | **Nadir Scr (µmol/L) after 1^st^ recurrence; follow-up (in months) after Tx** | **Discontinuation of Ecu** | **Second recurrence (time in months after Tx)** | **Restart ecu** | **Remarks** |  |
| [43] Fan et al. 2015 | No | 30 | LRD | *CFH:* p.Arg1251Glu | 21 days | Unknown | Unknown | Unknown | 199; specific time after Tx unknown | No | No | NA | - |  |
| [44]Iqbal et al.2015 | No | 49 | DD | *C3*: p.Asp1093Asn | 29 months | ±2 months | 100 | 396 | (±20 ml/min/1.73m2); ± 39 | No | No | NA | - |  |
| [11]Mallet et al.2015 | No | 40 | DD | Negative | Within 2 days | 2 days | Unknown | 620 (PT resistant) | Graft loss; 12 | NA | NA | NA | Graft loss 1 year after Tx due to anti-body mediated rejection, ongoing aHUS remission. |  |
| [11]Mallet et al.2015 | No | 23 | LRD | Unknown | 10 months | 4 months | Unknown | 450 (PT dependent) | 328; specific time after Tx unknown | No | NA | NA |  |  |
| [11]Mallet et al.2015 | No | 36 | DD | Negative | 5 years | 9.1 months | 80-90 | 130 (PT resistant) | 469; specific time after Tx unknown | NA | NA | NA | No renal or hematological respons to ecu. Visual and GI symptoms attributed to aHUS. Pt died 114 days after start of Ecu. |  |
| **Reference** | **aHUS in previous transplant** | **Recipients age (yrs) at Tx** | **Donor source** | **Reported genetic variant** | **Time from Tx to recurrence** | **Time from recurrence to start of Ecu** | **SCr ( µmol/L) (MDRD) before aHUS** | **Scr (µmol/L) (MDRD) at start of ecu** | **Nadir Scr (µmol/L) after 1^st^ recurrence; follow-up (in months) after Tx** | **Discontinuation of Ecu** | **Second recurrence (time in months after Tx)** | **Restart ecu** | **Remarks** |  |
| [45]Garlo et al.2015 | No | 30 | LURD | Unknown | 2 years | Unknown (after 9 sessies of PT) | 71 | 618 | 71; specific time after Tx unknown (two week after start of ecu) | Yes | Yes | Yes | Patient presented 26 weeks pregnant with pre-eclampsia for which a caesarian section was performed. Two days later she developed aHUS recurrence. Scr after 2^nd^ recurrence ± 106 µmol/L ± 3 years after Tx |  |
| [46]Broeders et al. 2014 | Yes | 41 | DCD | *CFH*: p.His860His | 6 months | 1 week | 141 | 168 (respons to PT unknown) | 141; 18 | No | NA | NA | 13 days after Tx also episode of TMA diagnosed as TMA due to ABMR, treated with steroids, IVIG and 3 sessions of PT leading to full recovery of kidney function |  |
| **Reference** | **aHUS in previous transplant** | **Recipients age (yrs) at Tx** | **Donor source** | **Reported genetic variant** | **Time from Tx to recurrence** | **Time from recurrence to start of Ecu** | **SCr ( µmol/L) (MDRD) before aHUS** | **Scr (µmol/L) (MDRD) at start of ecu** | **Nadir Scr (µmol/L) after 1^st^ recurrence; follow-up (in months) after Tx** | **Discontinuation of Ecu** | **Second recurrence (time in months after Tx)** | **Restart ecu** | **Remarks** |  |
| [19]Matar et al.2014 | Yes | 38 | LRD | *CFH*:p.Leu1189Phe | 6 years | Unknown | Unknown | Unknown | Unknown | Yes | Yes (84) | Yes | After 2^nd^ recurrence life-long Ecu therapy. Graft is still functioning. |  |
| [19]Matar et al.2014 | Yes | 33 | LURD | Negative | 3 months | Unknown | Unknown | Unknown | Unknown | Yes | Yes (17) | Yes | After restart of Ecu respons was seen. At 23 months after Tx graft loss due to contrast nephropathy and ATN. |  |
| [19]Matar et al.2014 | Yes | 57 | DD | *CFH*: p. Ile059Thre  *CFH*: p.Gln1143Glu | 3 months | Unknown | Unknown | Unknown | Graft loss; 6 | NA | NA | NA | Graft loss due to aHUS recurrence , no respons to Ecu. |  |
| [47]Commereuc et al.2013 | No | 22 | DD (combined lung-kidney) | Negative | 3 days | 12 days | 72 | HD | Unknown (*“hemodialysis could be stopped”)* | Unknown | No | NA | Unknown recovery of renal function, yet hemodialysis could be stopped. |  |
| **Reference** | **aHUS in previous transplant** | **Recipients age (yrs) at Tx** | **Donor source** | **Reported genetic variant** | **Time from Tx to recurrence** | **Time from recurrence to start of Ecu** | **SCr ( µmol/L) (MDRD) before aHUS** | **Scr (µmol/L) (MDRD) at start of ecu** | **Nadir Scr (µmol/L) after 1^st^ recurrence; follow-up (in months) after Tx** | **Discontinuation of Ecu** | **Second recurrence (time in months after Tx)** | **Restart ecu** | **Remarks** |  |
| [48]Sinibaldi et al.2013 | Yes | 19 | DD | *THBD*: p.Pro501Leu | 11 weeks | 5 days | ± 88 | ± 177 | ± 110-120; 4.5 | No | NA | NA | Graft loss 7 months after Tx due to cellular rejection unresponsive to steroids. |  |
| [49]Reuter et al.2013 | No | 24 | LRD | *MCP*: p.Tyr54Cys | 9 days | ± 14 days | 159 | ± 290 | 159; 44 | No | NA | NA | - |  |
| [50]Alachkar et al.2012 | Yes | 32 | LD | Negative | 3.5 months | Unknown (after 8 sessions of PT) | Unknown (*“excellent function”)* | HD | 159; 8 | Yes | Yes (13) | Yes | Scr after 2^nd^ recurrence 282 µmol/L. Graft loss, 2 years after Tx, due to complicated endovascular procedure with ATN, no signs of TMA in peripheral blood. |  |
| **Reference** | **aHUS in previous transplant** | **Recipients age (yrs) at Tx** | **Donor source** | **Reported genetic variant** | **Time from Tx to recurrence** | **Time from recurrence to start of Ecu** | **SCr ( µmol/L) (MDRD) before aHUS** | **Scr (µmol/L) (MDRD) at start of ecu** | **Nadir Scr (µmol/L) after 1^st^ recurrence; follow-up (in months) after Tx** | **Discontinuation of Ecu** | **Second recurrence (time in months after Tx)** | **Restart ecu** | **Remarks** |  |
| [22] Zuber et al. 2012 | Yes | Unknown | Unknown | *CFHR1-3* del  Anti *CFH* ab | 5 years | 3 months | Unknown | 89 (incomplete respons PT) | 80; ± 72 | No | Yes, relapse while on eculizumab therapy (± 86) | NA | During Ecu therapy fresh TMA lesions on renal biopsy, possible also due to chronic active ABMR. CH50 below detection threshold. Unknown renal function after 2^nd^ relapse. |  |
| [22] Gueutin by Zuber et al. 2012 | Yes | Unknown | Unknown | *CFI:* p. Gly101Arg | ± 36 months | 9 weeks | ± 156 | 190 (PT resistant) | Unknown | No | Yes, relapse while on eculizumab therapy |  | During Ecu therapy TMA in peripheral blood after interval prolongation. Scr after 2^nd^ relapse 156 µmol/L ± 40,5months after Tx. |  |
| **Reference** | **aHUS in previous transplant** | **Recipients age (yrs) at Tx** | **Donor source** | **Reported genetic variant** | **Time from Tx to recurrence** | **Time from recurrence to start of Ecu** | **SCr ( µmol/L) (MDRD) before aHUS** | **Scr (µmol/L) (MDRD) at start of ecu** | **Nadir Scr (µmol/L) after 1^st^ recurrence; follow-up (in months) after Tx** | **Discontinuation of Ecu** | **Second recurrence (time in months after Tx)** | **Restart ecu** | **Remarks** |  |
| [22] Zuber et al. 2012 | Yes | Unknown | Unknown | *CFH*: p.Ser1191Leu  *CFH*: p.Val1197Ala | 3 days | 3 days | Unknown | 627 (PT resistant) | 65; 17 | No | NA | NA | - |  |
| [22] Zuber et al. 2012 | Yes | Unknown | Unknown | *CFH-CRFH1* hybrid gene | 3 days | 1 month | Unknown | 237 (incomplete respons PT) | Unknown | No | Yes, relapse while on eculizumab therapy (± 4) | NA | During eculizumab therapy fresh TMA lesions on renal biopsy, possible partly due to high tacrolimus trough levels. CH50 below detection threshold. Serum creatinine after 2^nd^ relapse 204 µmol/L ± 15 months after Tx. |  |
| [51]Zlamy et al.2012 | No | 10 | DD | *CFH* | 6 days | 4 days | Unknown | GFR 45 ml/min/m² | 48; 27 | No | NA | NA | - |  |
| [52]Hodgkins et al.2012 | No | 5 | DD | Negative | 4 years and 6 months | 2 days | 44-53 | 751 | ± 53; 84 | No | NA | NA | - |  |
| **Reference** | **aHUS in previous transplant** | **Recipients age (yrs) at Tx** | **Donor source** | **Reported genetic variant** | **Time from Tx to recurrence** | **Time from recurrence to start of Ecu** | **SCr ( µmol/L) (MDRD) before aHUS** | **Scr (µmol/L) (MDRD) at start of ecu** | **Nadir Scr (µmol/L) after 1^st^ recurrence; follow-up (in months) after Tx** | **Discontinuation of Ecu** | **Second recurrence (time in months after Tx)** | **Restart ecu** | **Remarks** |  |
| [22,53]Duran et al. 2012 | No | 32 | DCD | *CFH:* p.Glu1172Stop | 2.5 months | 1 month | 159 | Hemodialysis | 168; ± 20 | No | NA | NA | - |  |
| [22,54]Heyne et al. 2011 | Yes | Unknown | Unknown | Not investigated | 8 days | 1 day | Unknown | 176 | 114; specific time after Tx unknown | Yes | Yes (±11) | Yes | Scr after 2^nd^ relapse 123 µmol/L; ± 25 months after Tx |  |
| [55]Wilson et al.2011 | No | 46 | DD (pancreas-kidney) | Negative | 41 days | 3 weeks | Unknown | Unknown | (40 ml/min/1.73m²); 24 | Yes | No | NA | - |  |
| [56]Chandran et al.2011 | No | 34 | DD (pancreas-kidney) | Unknown | 10 days | 4 days | 88 | HD | 63; 4 | Yes | No | No | - |  |
| [22,57]Al-Akash et al. 2010 | Yes | 15 | DD | *C3:* p.Arg570Trp | 9 weeks | 3 weeks | 115 | 202 (incomplete respons PT) | 115; ±42 | No | NA | NA | - |  |
| [22,58]Larrea et al. 2010 | No | 22 | DCD | Negative | 12 days | 9 days | 97 | 415 (PT resistant) | 70; specific time after Tx unknown | Yes | Yes (11) | Yes | Discontinuation after 1 dosage of ecu; after 2^nd^ relapse Scr of 175 µmol/L. After 4 months Ecu was stopped because of infection; GL loss due to ABMR. |  |
| **Reference** | **aHUS in previous transplant** | **Recipients age (yrs) at Tx** | **Donor source** | **Reported genetic variant** | **Time from Tx to recurrence** | **Time from recurrence to start of Ecu** | **SCr ( µmol/L) (MDRD) before aHUS** | **Scr (µmol/L) (MDRD) at start of ecu** | **Nadir Scr (µmol/L) after 1^st^ recurrence; follow-up (in months) after Tx** | **Discontinuation of Ecu** | **Second recurrence (time in months after Tx)** | **Restart ecu** | **Remarks** |  |
| [25,59]Châtelet et al.2010 | Yes | >36 | DD | *C3:* p.Arg570Gln | 4 years | 5 weeks | ± 160 | 320 (PT dependent) | Unknown | No | Yes, 2 x mild relapse while on eculizumab | NA | First aHUS recurrence after current Tx was successfully treated with PE. Second recurrence is described here. During Ecu therapy TMA in peripheral blood after interval prolongation. CH50 below detection threshold. Serum creatinine after relapse 230 µmol/L 68 months after Tx. |  |
| [25,60]Davin et al.2010 | Yes | 17 | Unknown | *CFH*: p.Ser1191Leu | 4 months | 6 months | 130 | 131 (PT dependent) | 120; ± 49 | No | NA | NA | - |  |
| **Reference** | **aHUS in previous transplant** | **Recipients age (yrs) at Tx** | **Donor source** | **Reported genetic variant** | **Time from Tx to recurrence** | **Time from recurrence to start of Ecu** | **SCr ( µmol/L) (MDRD) before aHUS** | **Scr (µmol/L) (MDRD) at start of ecu** | **Nadir Scr (µmol/L) after 1^st^ recurrence; follow-up (in months) after Tx** | **Discontinuation of Ecu** | **Second recurrence (time in months after Tx)** | **Restart ecu** | **Remarks** |  |
| [22]Ardissino et al. 2010 | No | Unknown | Unknown | *CFH* | 2 months | 2 days | Unknown | 442 (incomplete respons PT) | 48; ± 27 | No | NA | NA | - |  |
| [22,61]Legault et al. 2009 | No | 34 | LRD | Unknown | 1 month | ± 8 months | 106 | 321 (incomplete respons PT) | 238; ± 15 | No | NA | NA | - |  |
| [22,62]Nürnberger et al.2009 | Yes | 37 | DD | *CFH*: p.Tyr475Ser | 6 weeks | 5 days | ±107 | 132 (PT resistant) | 97; specific time after Tx unknown | Yes | Yes (21) | No | Discontinuation after 1 dosage of Ecu; second recurrence, not treated with eculizumab, led to graft loss 22 months after Tx. |  |

*Case reports and case series found in literature with the use of eculizumab in case of aHUS recurrence after kidney transplantation. In several case reports serum creatinine (or follow up time) is not described in text but depicted in a figure, the approximate values were used in this table and designated as ± . For the ten patients indicated in red a serum creatinine before the onset of aHUS and after the recurrence was available and these patients had started eculizumab therapy within 28 days after onset of aHUS. Three patients (indicated with *) were excluded as these patients had a poor renal function before the onset of aHUS due of infectious complications or a rejection.*

*Abbreviations: LRD= living related donor; LD= living donor; DD= deceased donor; DBD= donation after brain death; DCD= donation after cardiac death; LURD= living unrelated donor; ABMR= antibody mediated rejection; CFH= complement factor H; CFI= complement factor I; CFB=complement factor B; C3= complement factor 3; MCP= membrane cofactor protein; THBD= thrombomodulin; CFHR= complement factor H related protein; del= deletion; Tx = kidney transplantation; MDRD = modification of diet in renal disease; ABOi= ABO incompatible; anti CFH ab= anti CFH antibodies; Ecu=eculizumab; NA= not applicable; Bx=kidney biopsy; TMA = thrombotic microangiopathy; PE = plasma exchange; GL =graft loss; ATN = acute tubular necrosis; GI = gastro-intestinal; IVIG = intravenous immunoglobulins; Scr= serum creatinine; HD = hemodialysis; iCVA = ischemic cerebrovasculair accident; FU = follow-up.*

*¹individual improvement in kidney function is depicted in a figure in article by Andrade et al. The mean serum creatinine for all 5 patients was 362 µmol/L ± 62 µmol/L at start of eculizumab and 168 µmol/L ± 53 µmol/L after 6 months of treatment with eculizumab.*

*² Eculizumab was discontinued 170 days after the second recurrence. A third recurrence was suspected based on slow increase in serum creatinine and confirmed, almost a year after discontinuation, by kidney biopsy. No signs of peripheral blood TMA were seen. Eculizumab was restarted and improvement of kidney function was seen.*

**Online resource table 3: characteristics and outcome of rescue therapy according to genetics in aHUS renal transplantation patients**

| Mutation | aHUS in previous transplant | Donor source (%) | Median Time from Tx to recurrence in days (range) | Start of Ecu ≤ 7 days after recurrence | Time from recurrence to Ecu in days (range) | Nadir median Scr (µmol/L) after 1^st^ recurrence ²  (range) | Discontinuation of Ecu | Second recurrence after discontinuation | Graft loss (due to aHUS) |
| --- | --- | --- | --- | --- | --- | --- | --- | --- | --- |
| CFH mutation (including 3 patients with CFH/CFHR hybrid genes)  (N=19)  [5-7,19,25,31,35,40,41,43,46,51,53,60,62] | 9 (47%) | 10 (53%) DD  4 (21%) LD  5 (26%) unknown | 42 (0-2190) | 9 (47%) | 5 (1-244) | 110 (48-199) | 4 (21%) | 4 (100%) | 3/19 (2) ³ |
| Other mutation¹  (N=13)  [6,7,25,29,30,32,36,44,48,49,57,63] | 6 (46%) | 7 (54%) DD  3 (23%) LD  3 (23%) unknown | 77 (9-1825) | 3 (23%) | 21 (0-120) | 154 (80-486) | 2 (15%) | 1 (50%) | 1/13 (0) |
| Negative  (N=13)  [6,11,19,34,38,42,47,50,52,55,58] | 2 (15%) | 8 (62%) DD  5 (38%) LD | 41 (2-1825) | 4 (31%) | 10 (2-279) | 150 (53-185) | 5 (42%) | 3 (60%) | 4/13 (0) |
| Unknown  (N=11)  [6,7,11,33,39,45,54,56,61] | 0 (0%) | 3 (27%) DD  5 (45%) LD  3 (27%) unknown | 7 (0-730) | 5 (45%) | 5 (1-244) | 120 (63-328) | 6 (50%) | 3 (50%) | 1/11 (1) |
| P- value⁴ | P=0.075 | P=0.313 | P=0.016 | P=0.889 | P=0.628 | P=0.156 |  |  |  |

*Characteristics and outcome of rescue therapy according to genetic mutation. The patients are divided into groups: CFH or CFH/CFHR hybrid gene, other mutation, no mutation or unknown mutational status.*

*Abbreviations: LD= living donor; DD= deceased donor; CFH= complement factor H; CFI= complement factor I; CFB=complement factor B; C3= complement factor 3; MCP= membrane cofactor protein; THBD= thrombomodulin; CFHR= complement factor H related protein; del= deletion; Tx = kidney transplantation; MDRD = modification of diet in renal disease; ABOi= ABO incompatible; anti CFH ab= anti CFH antibodies; Ecu=eculizumab; Scr= serum creatinine.*

*¹Other mutation: 4 mutations in the gene coding for CFI, 2 mutations in the gene coding for MCP, 4 mutations in the gene coding for C3, 2 CFHR1-3 deletions with anti-CFH antibodies, 1 mutation in the gene coding for THBD and 4 patients with CFHR1-3 deletions without anti CFH antibodies.*

*² Excluding the patients who died (N=1), suffered from graft loss (N=9) and the patients without a reported nadir serum creatinine (N=11).*

*³ Two patients lost their grafts due to aHUS. One patient had not responded to eculizumab treatment and the second patient discontinued eculizumab therapy after one dose, leading to a second recurrence, which was not treated with eculizumab. [7,62]*

*⁴Statistical method: Kruskal-Wallis test*

**Online resource table 4: characteristics and outcome of prophylactic therapy according to genetics in aHUS renal transplantation patients**

| Mutation | aHUS in previous transplant | Donor source (%) | Discontinuation of Ecu ² | Recurrence after discontinuation | Graft loss (due to aHUS) |
| --- | --- | --- | --- | --- | --- |
| CFH mutation (including 3 patients with CFH/CFHR hybrid genes)  (N=25)  [1,3,5,6,8-10,12-14,17-19,21,23-28] | 4 (16%) | 14 (56%) DD  11(44%) LD | 2 (8%) | 1 (50%) | 2 (1) |
| Other mutation¹  (N=14)  [2,4-6,12,15,20,25,64] | 2 (14%) | 11(79%) DD  2 (14%) LD  1 (7%) unknown | 1 (7%) | 0 (0%) | 0³ |
| Negative  (N=5)  [2,11,19] | 3 (60%) | 0 (0%) DD  5 (100%) LD | 4 (80%) | 1 (25%) | 0 |
| Unknown  (N=9)  [5,7] | 0 (0%) | 1(1%) DD  0 (0%) LD  8 (89%) unknown | 1(11%) | 0 (0%) | 0 |
| P- value⁴ | P=0.000 | P=0.000 | P=0.000 | P=0.761 | P=1.000 |

*Characteristics and outcome of prophylactic therapy according to genetic mutation. The patients are divided into groups: CFH or CFH/CFHR hybrid gene, other mutation, no mutation or unknown mutational status.*

*Abbreviations: LD= living donor; DD= deceased donor; CFH= complement factor H; CFI= complement factor I; CFB=complement factor B; C3= complement factor 3; MCP= membrane cofactor protein; THBD= thrombomodulin; CFHR= complement factor H related protein; del= deletion; Tx = kidney transplantation; MDRD = modification of diet in renal disease; ABOi= ABO incompatible; anti CFH ab= anti CFH antibodies; Ecu=eculizumab; Scr= serum creatinine.*

*¹Other mutation: 5 mutations in the gene coding for CFI, 1 mutation in the gene coding for CFB, 1 mutation in the gene coding for MCP, 3 mutations in the gene coding for C3, 3 patients with anti CFH antibodies, 1 mutation in the gene coding for THBD and 2 other mutations in the genes coding for CFHR.*

*² The amount of patients who discontinued eculizumab (not according to donor source).*

*³ One patient died of a cerebrovascular incident, possibly an extrarenal manifestation of aHUS. [15]*

*⁴ Statistical method: Kruskal-Wallis test*

**Online resource table 5: Kidney transplantation protocol to minimize endothelial injury in aHUS patients**

| **Treatment** | **Dosage / targets** |
| --- | --- |
| Living donor kidney |  |
| Basiliximab | 20 mg day 1 and day 4 |
| Tacrolimus | Starting dose 0.03 mg/kg twice daily; target blood levels of 4-5 µg/L first 30 days, thereafter 5-7 µg/L |
| Prednisone | Starting dose 100 mg/day on days 1-3, thereafter 25 mg/day and tapering to 0.1 mg/kg/day at 3 months after transplantation |
| Mycophenolate mofetil | Starting dose 1000 mg twice daily; target area under the curve 40-60 mg/mL/hour |
| Blood pressure | Target <130/80 mmHg |
| Early introduction of ACE inhibitor |  |
| Early introduction of statins |  |

*Transplantation protocol for adults aHUS patients aimed to minimize endothelial injury. This protocol was used previously by Duineveld et al. and Verhave et al.[35,65]. Transplantation from a living related donor can be considered if the causative genetic factor is identified in the recipient and is absent in the donor [66]. After transplantation close monitoring is advised: daily for the first 2 week, twice weekly for the next 2 to 4 weeks and weekly thereafter until 4 months after transplantation, followed by gradual extension of the interval to every 6 weeks at 12 months and every 3 months after 2 years. Home blood pressure measurements are advised.*

**Online resource table 6: Proposed guidance for the selection of restrictive treatment scenarios based on patient characteristics**

| **Characteristics** |  | **Points** |
| --- | --- | --- |
| **Age** | ≤ 6 | 5 |
|  | 6-12 years | 1 |
|  | > 12 years | 0 |
| **Native kidney** |  |  |
| First episode |  | 0 |
| Any relapse , never treated with eculizumab |  | 0 |
| First relapse >12 months after eculizumab withdrawal |  | 1 |
| First relapse >3- <12 months after eculizumab withdrawal |  | 2 |
| First relapse <3 months after eculizumab withdrawal |  | 4 |
| Multiple relapses after eculizumab withdrawal |  | 3 |
| Relapse while on eculizumab (with incomplete complement blockade) |  | 4 |
| Relapse while on eculizumab (with complete complement blockade) |  | 14 |
| **Kidney transplantation** |  |  |
| First episode of recurrence > 1-3 months after transplantation |  | 2 |
| First episode of recurrence < 1-3 months after transplantation |  | 3 |
| Second relapse > 12 months after eculizumab withdrawal |  | 3 |
| Second relapse < 12 months after eculizumab withdrawal |  | 4 |
| Relapse while on eculizumab (with incomplete complement blockade) |  | 4 |
| Relapse while on eculizumab (with complete complement blockade) |  | 14 |
| Having received prophylactic treatment because of anticipated high risk |  | 8 |
| **Genetic analysis** |  |  |
| No mutation |  | 0 |
| Mutation not in complement factor H |  | 1 |
| Mutation in complement factor H |  | 2 |

*To assist in the selection of a certain scenariowe have tried to describe patient characteristics and attribute a score; points can be added for age, aHUS in a native kidney or allograft and for the presence of a genetic mutation. The total score (sum of points)provides guidance forthe selection of a scenario, however based on additional clinical characteristics and/or physicians or patients preference a different choice can be made. A total score of 1 points corresponds with scenario 1, a total score of 2 points corresponds with scenario 2, a total score of 3 points corresponds with scenario 3, a total score of 4 points corresponds with scenario 4, a total score of 5-13 points corresponds to scenario 5 and a score >13 points corresponds with scenario 6.*

**References online resource tables:**

1. Nieto-Rios JF, Zuluaga-Quintero M, Bello-Marquez DC, Aristizabal-Alzate A, Ocampo-Kohn C, Serna-Higuita LM, Arias L, Zuluaga-Valencia G (2018) Successful kidney transplant with eculizumab, thymoglobulin and belatacept therapy in a highly-sensitised patient with atypical haemolytic uraemic syndrome due to factor H mutation. Nefrologia10.1016/j.nefro.2017.09.013

2. Macia M, de Alvaro Moreno F, Dutt T, Fehrman I, Hadaya K, Gasteyger C, Heyne N (2017) Current evidence on the discontinuation of eculizumab in patients with atypical haemolytic uraemic syndrome. Clin Kidney J 10:310-319 doi:10.1093/ckj/sfw115

3. Krishnan AR, Siva B, Chakera A, Wong G, Wong D, Lim WH (2017) Absence of thrombocytopaenia and/or microangiopathic haemolytic anaemia does not reliably exclude recurrence of atypical haemolytic uraemic syndrome after kidney transplantation. Nephrology 22:28-31 doi:10.1111/nep.12937

4. Manani SM, Virzi GM, Giuliani A, Clementi A, Brocca A, Dissegna D, Martino F, d'Amore ESG, Ronco C (2017) Hemolytic Uremic Syndrome and Kidney Transplantation: A Case Series and Review of the Literature. Nephron 136:245-253 doi:10.1159/000468528

5. Levi C, Fremeaux-Bacchi V, Zuber J, Rabant M, Devriese M, Snanoudj R, Scemla A, Amrouche L, Mejean A, Legendre C, Sberro-Soussan R (2017) Midterm Outcomes of 12 Renal Transplant Recipients Treated With Eculizumab to Prevent Atypical Hemolytic Syndrome Recurrence. Transplantation 101:2924-2930 doi:10.1097/tp.0000000000001909

6. de Andrade LGM, Contti MM, Nga HS, Bravin AM, Takase HM, Viero RM, da Silva TN, Chagas KN, Palma LMP (2017) Long-term outcomes of the Atypical Hemolytic Uremic Syndrome after kidney transplantation treated with eculizumab as first choice. PLoS One 12:e0188155 doi:10.1371/journal.pone.0188155

7. Sheerin NS, Kavanagh D, Goodship TH, Johnson S (2016) A national specialized service in England for atypical haemolytic uraemic syndrome-the first year's experience. QJM 109:27-33 doi:10.1093/qjmed/hcv082

8. Sun ZJ, Du X, Su LL, Zhang XD, Wang Y, Ren L, Wang W (2016) Successful Renal Transplantation in a Patient with Atypical Hemolytic Uremic Syndrome Treated with Eculizumab in China. Chin Med J (Engl) 129:1379-1381 doi:10.4103/0366-6999.182843

9. Akchurin O, Dogra S, Kaskel F, Jan D, Greenstein S, Del Rio M (2016) Preemptive use of eculizumab for living-donor kidney transplantation in a child with atypical hemolytic uremic syndrome. Einstein Journal of Biology and Medicine 30:22-25 doi:

10. Kasapoglu U, Ruhi C, Tugcu M, Boynuegri B, Titiz I, Hancer VS, Apaydin S (2015) Prophylactic Eculizumab Use in Kidney Transplantation: A Review of the Literature and Report of a Case with Atypical Hemolytic Uremic Syndrome. Ann Transplant 20:714-719 doi:

11. Mallett A, Hughes P, Szer J, Tuckfield A, Van Eps C, Cambell SB, Hawley C, Burke J, Kausman J, Hewitt I, Parnham A, Ford S, Isbel N (2015) Atypical haemolytic uraemic syndrome treated with the complement inhibitor eculizumab: the experience of the Australian compassionate access cohort. Intern Med J 45:1054-1065 doi:10.1111/imj.12864

12. Alasfar S, Alachkar N (2014) Atypical hemolytic uremic syndrome post-kidney transplantation: two case reports and review of the literature. Front Med (Lausanne) 1:52 doi:10.3389/fmed.2014.00052

13. Riddell A, Goodship T, Bingham C (2016) Prevention of recurrence of atypical hemolytic uremic syndrome post renal transplant with the use of higher-dose eculizumab. Clin Nephrol 86:200-202 doi:10.5414/cn108808

14. Parikova A, Fronek JP, Viklicky O (2015) Living-donor kidney transplantation for atypical haemolytic uremic syndrome with pre-emptive eculizumab use. Transpl Int 28:366-369 doi:10.1111/tri.12440

15. Azukaitis K, Loirat C, Malina M, Adomaitiene I, Jankauskiene A (2014) Macrovascular involvement in a child with atypical hemolytic uremic syndrome. Pediatr Nephrol 29:1273-1277 doi:10.1007/s00467-013-2713-3

16. Ardissino G, Possenti I, Tel F (2015) In reply to 'discontinuation of eculizumab maintenance treatment for atypical hemolytic uremic syndrome'. Am J Kidney Dis 65:342-343 doi:10.1053/j.ajkd.2014.05.028

17. Ranch D, Crowther B, Arar M, Assanasen C (2014) Prophylactic eculizumab for kidney transplantation in a child with atypical hemolytic uremic syndrome due to complement factor H mutation. Pediatr Transplant 18:E185-189 doi:10.1111/petr.12290

18. Roman-Ortiz E, Mendizabal Oteiza S, Pinto S, Lopez-Trascasa M, Sanchez-Corral P, Rodriguez de Cordoba S (2014) Eculizumab long-term therapy for pediatric renal transplant in aHUS with CFH/CFHR1 hybrid gene. Pediatr Nephrol 29:149-153 doi:10.1007/s00467-013-2591-8

19. Matar D, Naqvi F, Racusen LC, Carter-Monroe N, Montgomery RA, Alachkar N (2014) Atypical hemolytic uremic syndrome recurrence after kidney transplantation. Transplantation 98:1205-1212 doi:10.1097/tp.0000000000000200

20. Bekassy ZD, Kristoffersson AC, Cronqvist M, Roumenina LT, Rybkine T, Vergoz L, Hue C, Fremeaux-Bacchi V, Karpman D (2013) Eculizumab in an anephric patient with atypical haemolytic uraemic syndrome and advanced vascular lesions. Nephrol Dial Transplant 28:2899-2907 doi:10.1093/ndt/gft340

21. Pelicano MB, de Cordoba SR, Diekmann F, Saiz M, Herrero S, Oppenheimer F, Campistol JM (2013) Anti-C5 as prophylactic therapy in atypical hemolytic uremic syndrome in living-related kidney transplantation. Transplantation 96:e26-29 doi:10.1097/TP.0b013e31829d388d

22. Zuber J, Fakhouri F, Roumenina LT, Loirat C, Fremeaux-Bacchi V (2012) Use of eculizumab for atypical haemolytic uraemic syndrome and C3 glomerulopathies. Nat Rev Nephrol 8:643-657 doi:10.1038/nrneph.2012.214

23. Xie L, Nester CM, Reed AI, Zhang Y, Smith RJ, Thomas CP (2012) Tailored eculizumab therapy in the management of complement factor H-mediated atypical hemolytic uremic syndrome in an adult kidney transplant recipient: a case report. Transplant Proc 44:3037-3040 doi:10.1016/j.transproceed.2012.07.141

24. Krid S, Roumenina LT, Beury D, Charbit M, Boyer O, Fremeaux-Bacchi V, Niaudet P (2012) Renal transplantation under prophylactic eculizumab in atypical hemolytic uremic syndrome with CFH/CFHR1 hybrid protein. Am J Transplant 12:1938-1944 doi:10.1111/j.1600-6143.2012.04051.x

25. Zuber J, Le Quintrec M, Krid S, Bertoye C, Gueutin V, Lahoche A, Heyne N, Ardissino G, Chatelet V, Noel LH, Hourmant M, Niaudet P, Fremeaux-Bacchi V, Rondeau E, Legendre C, Loirat C (2012) Eculizumab for atypical hemolytic uremic syndrome recurrence in renal transplantation. Am J Transplant 12:3337-3354 doi:10.1111/j.1600-6143.2012.04252.x

26. Nester C, Stewart Z, Myers D, Jetton J, Nair R, Reed A, Thomas C, Smith R, Brophy P (2011) Pre-emptive eculizumab and plasmapheresis for renal transplant in atypical hemolytic uremic syndrome. Clin J Am Soc Nephrol 6:1488-1494 doi:10.2215/cjn.10181110

27. Weitz M, Amon O, Bassler D, Koenigsrainer A, Nadalin S (2011) Prophylactic eculizumab prior to kidney transplantation for atypical hemolytic uremic syndrome. Pediatr Nephrol 26:1325-1329 doi:10.1007/s00467-011-1879-9

28. Zimmerhackl LB, Hofer J, Cortina G, Mark W, Wurzner R, Jungraithmayr TC, Khursigara G, Kliche KO, Radauer W (2010) Prophylactic eculizumab after renal transplantation in atypical hemolytic-uremic syndrome. N Engl J Med 362:1746-1748 doi:10.1056/NEJMc1001060

29. Devresse A, de Meyer M, Aydin S, Dahan K, Kanaan N (2018) De Novo Atypical Haemolytic Uremic Syndrome after Kidney Transplantation. Case Rep Nephrol 2018:1727986 doi:10.1155/2018/1727986

30. Zwang NA, Ho B, Kanwar YS, Lewis B, Cusick M, Friedewald JJ, Gallon L (2018) A case of atypical hemolytic uremic syndrome in a second renal transplant. J Nephrol 31:165-172 doi:10.1007/s40620-016-0373-7

31. Vondrak K, Seeman T (2018) Successful 7-Year Eculizumab Treatment of Plasmapheresis-Resistant Recurrent Atypical Hemolytic-Uremic Syndrome due to Complement Factor H Hybrid Gene: A Case Report. Transplant Proc 50:967-970 doi:10.1016/j.transproceed.2018.02.012

32. Munch J, Bachmann A, Grohmann M, Mayer C, Kirschfink M, Lindner TH, Bergmann C, Halbritter J (2017) Effective immunosuppressive management with belatacept and eculizumab in post-transplant aHUS due to a homozygous deletion of CFHR1/CFHR3 and the presence of CFH antibodies. Clinical Kidney Journal 10:742-746 doi:10.1093/ckj/sfx053

33. Shochet L, Kanellis J, Simpson I, Ta J, Mulley W (2017) De novo thrombotic microangiopathy following simultaneous pancreas and kidney transplantation managed with eculizumab. Nephrology 22:23-27 doi:10.1111/nep.12936

34. Asif A, Nayer A, Haas CS (2017) Atypical hemolytic uremic syndrome in the setting of complement-amplifying conditions: case reports and a review of the evidence for treatment with eculizumab. Journal of Nephrology 30:347-362 doi:10.1007/s40620-016-0357-7

35. Duineveld C, Verhave JC, Berger SP, van de Kar N, Wetzels JFM (2017) Living Donor Kidney Transplantation in Atypical Hemolytic Uremic Syndrome: A Case Series. Am J Kidney Dis 70:770-777 doi:10.1053/j.ajkd.2017.06.024

36. Dedhia P, Govil A, Mogilishetty G, Alloway RR, Woodle ES, Abu Jawdeh BG (2017) Eculizumab and Belatacept for De Novo Atypical Hemolytic Uremic Syndrome Associated With CFHR3-CFHR1 Deletion in a Kidney Transplant Recipient: A Case Report. Transplant Proc 49:188-192 doi:10.1016/j.transproceed.2016.11.008

37. Legendre CM, Campistol JM, Feldkamp T, Remuzzi G, Kincaid JF, Lommele A, Wang J, Weekers LE, Sheerin NS (2017) Outcomes of patients with atypical haemolytic uraemic syndrome with native and transplanted kidneys treated with eculizumab: a pooled post hoc analysis. Transpl Int 30:1275-1283 doi:10.1111/tri.13022

38. Yamamoto T, Watarai Y, Futamura K, Okada M, Tsujita M, Hiramitsu T, Goto N, Narumi S, Takeda A, Kobayashi T (2017) Efficacy of Eculizumab Therapy for Atypical Hemolytic Uremic Syndrome Recurrence and Antibody-Mediated Rejection Progress After Renal Transplantation With Preformed Donor-Specific Antibodies: Case Report. Transplant Proc 49:159-162 doi:10.1016/j.transproceed.2016.10.013

39. Ikeda T, Okumi M, Unagami K, Kanzawa T, Sawada A, Kawanishi K, Omoto K, Ishida H, Tanabe K (2016) Two cases of kidney transplantation-associated thrombotic microangiopathy successfully treated with eculizumab. Nephrology 21:35-40 doi:10.1111/nep.12768

40. Okumi M, Omoto K, Unagami K, Ishida H, Tanabe K (2016) Eculizumab for the treatment of atypical hemolytic uremic syndrome recurrence after kidney transplantation associated with complement factor H mutations: a case report with a 5-year follow-up. Int Urol Nephrol 48:817-818 doi:10.1007/s11255-016-1234-y

41. Salameh H, Abu Omar M, Alhariri A, Kisra S, Qasem A, Abdulhak AB (2016) Adult Post-Kidney Transplant Familial Atypical Hemolytic Uremic Syndrome Successfully Treated With Eculizumab: A Case Report and Literature Review. Am J Ther 23:e1110-1115 doi:10.1097/mjt.0000000000000133

42. Juega-Marino FJ, Sala N, Lopez D, Canas L, Bonet J, Lauzurica R (2016) Late onset of de novo atypical hemolytic-uremic syndrome presented on a simultaneous pancreas and kidney transplant recipient successfully treated with eculizumab. Nefrologia 36:328-329 doi:10.1016/j.nefro.2016.02.003

43. Fan J, Tryphonopoulos P, Tekin A, Nishida S, Selvaggi G, Amador A, Jebrock J, Weppler D, Levi D, Vianna R, Ruiz P, Tzakis A (2015) Eculizumab Salvage Therapy for Antibody-Mediated Rejection in a Desensitization-Resistant Intestinal Re-Transplant Patient. Am J Transplant 15:1995-2000 doi:10.1111/ajt.13183

44. Iqbal Z, Wood K, Carter V, Goodship TH, Brown AL, Sheerin NS (2015) Thrombotic Microangiopathy as a Cause of Chronic Kidney Transplant Dysfunction: Case Report Demonstrating Successful Treatment with Eculizumab. Transplantation Proceedings 47:2258-2261 doi:10.1016/j.transproceed.2015.08.004

45. Garlo K, Dressel D, Savic M, Vella J (2015) Successful eculizumab treatment of recurrent postpartum atypical hemolytic uremic syndrome after kidney transplantation. Clin Nephrol Case Stud 3:8-13 doi:10.5414/CNCS108491

46. Broeders EN, Stordeur P, Rorive S, Dahan K (2014) A 'silent', new polymorphism of factor H and apparent de novo atypical haemolytic uraemic syndrome after kidney transplantation. BMJ Case Rep 201410.1136/bcr-2014-207630

47. Commereuc M, Karras A, Amrein C, Boussaud V, Sberro-Soussan R, Guillemain R, Bacchi VF, Thervet E (2013) Successful treatment of acute thrombotic microangiopathy by eculizumab after combined lung and kidney transplantation. Transplantation 96:e58-59 doi:10.1097/TP.0b013e3182a7fccd

48. Sinibaldi S, Guzzo I, Piras R, Bresin E, Emma F, Dello Strologo L (2013) Post-transplant recurrence of atypical hemolytic uremic syndrome in a patient with thrombomodulin mutation. Pediatric Transplantation 17:E177-E181 doi:10.1111/petr.12151

49. Reuter S, Heitplatz B, Pavenstadt H, Suwelack B (2013) Successful long-term treatment of TMA with eculizumab in a transplanted patient with atypical hemolytic uremic syndrome due to MCP mutation. Transplantation 96:e74-76 doi:10.1097/01.TP.0000435705.63428.1f

50. Alachkar N, Bagnasco SM, Montgomery RA (2012) Eculizumab for the treatment of two recurrences of atypical hemolytic uremic syndrome in a kidney allograft. Transpl Int 25:e93-95 doi:10.1111/j.1432-2277.2012.01497.x

51. Zlamy M, Hofer J, Elias J, Vogel U, Frosch M, Jungraithmayr T, Zimmerhackl LB, Prelog M (2012) Immunogenicity of meningococcus C vaccination in a patient with atypical hemolytic uremic syndrome (aHUS) on eculizumab therapy. Pediatr Transplant 16:E246-E250 doi:10.1111/j.1399-3046.2011.01585.x

52. Hodgkins KS, Bobrowski AE, Lane JC, Langman CB (2012) Clinical Grand Rounds: Atypical Hemolytic Uremic Syndrome. American Journal of Nephrology 35:394-400 doi:10.1159/000337954

53. Duran CE, Blasco M, Maduell F, Campistol JM (2012) Rescue therapy with eculizumab in a transplant recipient with atypical haemolytic-uraemic syndrome. Clin Kidney J 5:28-30 doi:10.1093/ndtplus/sfr107

54. Heyne N, Weitz M, Guthoff M, Alscher M, Häring H-u, Königsrainer A, Nadalin S (2011) Terminal complement blockade by eculizumab effectively reverses recurrent atypical hemolytic uremic syndrome after kidney transplantation. Transpl Int 24:35 doi:

55. Wilson CH, Brown AL, White SA, Goodship THJ, Sheerin NS, Manas DM (2011) Successful Treatment of De Novo Posttransplant Thrombotic Microangiopathy With Eculizumab. Transplantation 92:E42-E43 doi:10.1097/TP.0b013e318230c0bd

56. Chandran S, Baxter-Lowe L, Olson JL, Tomlanovich SJ, Webber A (2011) Eculizumab for the Treatment of De Novo Thrombotic Microangiopathy Post Simultaneous Pancreas-Kidney Transplantation-A Case Report. Transplantation Proceedings 43:2097-2101 doi:10.1016/j.transproceed.2011.02.064

57. Al-Akash SI, Almond PS, Savell VH, Jr., Gharaybeh SI, Hogue C (2011) Eculizumab induces long-term remission in recurrent post-transplant HUS associated with C3 gene mutation. Pediatr Nephrol 26:613-619 doi:10.1007/s00467-010-1708-6

58. Larrea CF, Cofan F, Oppenheimer F, Campistol JM, Escolar G, Lozano M (2010) Efficacy of eculizumab in the treatment of recurrent atypical hemolytic-uremic syndrome after renal transplantation. Transplantation 89:903-904 doi:10.1097/TP.0b013e3181ccd80d

59. Chatelet V, Lobbedez T, Fremeaux-Bacchi V, Ficheux M, Ryckelynck JP, Hurault de Ligny B (2010) Eculizumab: safety and efficacy after 17 months of treatment in a renal transplant patient with recurrent atypical hemolytic-uremic syndrome: case report. Transplant Proc 42:4353-4355 doi:10.1016/j.transproceed.2010.09.125

60. Davin JC, Gracchi V, Bouts A, Groothoff J, Strain L, Goodship T (2010) Maintenance of kidney function following treatment with eculizumab and discontinuation of plasma exchange after a third kidney transplant for atypical hemolytic uremic syndrome associated with a CFH mutation. Am J Kidney Dis 55:708-711 doi:10.1053/j.ajkd.2009.08.011

61. Legault DJ, Boelkins MR (2009) Successful Treatment of aHUS Recurrence and Arrest of Plasma Exchange Resistant TMA Post-Renal Transplantation with the Terminal Complement Inhibitor Eculizumab. Am Soc Hematology,

62. Nurnberger J, Philipp T, Witzke O, Opazo Saez A, Vester U, Baba HA, Kribben A, Zimmerhackl LB, Janecke AR, Nagel M, Kirschfink M (2009) Eculizumab for atypical hemolytic-uremic syndrome. N Engl J Med 360:542-544 doi:10.1056/NEJMc0808527

63. Chatelet V, Fremeaux-Bacchi V, Lobbedez T, Ficheux M, Hurault de Ligny B (2009) Safety and long-term efficacy of eculizumab in a renal transplant patient with recurrent atypical hemolytic-uremic syndrome. Am J Transplant 9:2644-2645 doi:10.1111/j.1600-6143.2009.02817.x

64. Ardissino G, Possenti I, Tel F, Testa S, Salardi S, Ladisa V (2015) Discontinuation of eculizumab treatment in atypical hemolytic uremic syndrome: an update. Am J Kidney Dis 66:172-173 doi:10.1053/j.ajkd.2015.04.010

65. Verhave JC, Westra D, van Hamersvelt HW, van Helden M, van de Kar NC, Wetzels JF (2013) Living kidney transplantation in adult patients with atypical haemolytic uraemic syndrome. Neth J Med 71:342-347 doi:

66. Goodship TH, Cook HT, Fakhouri F, Fervenza FC, Fremeaux-Bacchi V, Kavanagh D, Nester CM, Noris M, Pickering MC, Rodriguez de Cordoba S, Roumenina LT, Sethi S, Smith RJ, Conference P (2017) Atypical hemolytic uremic syndrome and C3 glomerulopathy: conclusions from a "Kidney Disease: Improving Global Outcomes" (KDIGO) Controversies Conference. Kidney Int 91:539-551 doi:10.1016/j.kint.2016.10.005
